# Magnetotactic bacteria as biostimulants for enhancing the growth and yield of tomato and paddy under abiotic stress

**DOI:** 10.3389/fmicb.2026.1848546

**Published:** 2026-06-26

**Authors:** Shraddha Shirsat, M. A. Jayasri, K. Suthindhiran

**Affiliations:** Marine Biotechnology and Bioproducts Laboratory, Department of Biomedical Sciences, School of Biosciences and Technology, Vellore Institute of Technology, Vellore, Tamil Nadu, India

**Keywords:** *Magnetospirillum*, biofertilizer, tomato, paddy, iron deficiency, soil salinity, plant growth

## Abstract

Nutrient imbalances, soil salinity, and shrinking arable land threaten global food security, driving demand for sustainable biofertilizer alternatives to chemical inputs. Aquatic ecosystem-derived biofertilizers such as *Magnetospirillum gryphiswaldense* (MSR-1) are promising sustainable substitutes and show strong agricultural potential due to their stress tolerance, adaptability, and plant growth-promoting traits. This study investigated the ability of MSR-1 to enhance the growth and productivity of tomato and paddy under normal, iron-deficient, and saline conditions. MSR-1 was cultured in modified *Magnetospirillum* Growth Medium (MSGM) under microaerophilic conditions, with SEM confirming its spiral gram-negative morphology and successful, non-destructive colonization on tomato and paddy roots and leaves. Moreover, HR-LCMS profiling of root exudates identified chemoattractant compounds such as quinic acid, tryptophan, quercetin, glucosinolates, and strigolactones, promoting bacterial attachment. Further, *Magnetospirillum* liquid biofertilizer (MLB) was formulated from MSR-1 cultures (1.5 × 10^8^ cells/mL) and applied at 20–100% concentrations (25 mL/pot). Among the treatments, 20% MLB gave the best results under normal conditions, whereas 60% MLB was more effective under iron-deficient and saline stress conditions. In tomato, 20% MLB increased shoot length (73.5 cm), chlorophyll content (4.5 mg/g), and fruit yield (1066.95 g/plant). Under stress, 60% MLB improved fruit yield (760–800 g/plant) and boosted antioxidant enzymes (SOD 75 U/mg; CAT 15.5 U/mg). In paddy, 20% MLB enhanced shoot and root length (66.0 and 15.13 cm), while 60% MLB under stress increased growth, carbohydrates, proteins, amino acids, phenols, and antioxidant enzymes (SOD/CAT 49.63/19.83 U/mg). Overall, MSR-1 offers a sustainable, effective biofertilizer option for managing soil salinity and iron deficiency.

## 1 Introduction

Global agricultural productivity is increasingly strained by nutrient deficiencies, soil salinity, and declining arable land. At the same time, these challenges are becoming harder to manage because the global population is expected to reach nearly 10 billion by 2050, along with fast-paced urban growth (FAO, 2023). This had led to land overexploitation due to excessive use of chemical fertilizers, causing nutrient imbalances and soil degradation, reducing long-term crop productivity. Globally, the use of inorganic fertilizers reached nearly 185 million tonnes in 2022, demonstrating the scale of their application (FAO, 2023). The improper use of chemical fertilizers contributes to soil salinity. Soil salinity affects about 20% of the world’s irrigated land and 6% of all land globally (Wang et al., 2024). This results in significant yield losses, with at least 25% of crops experiencing salt stress (Ma et al., 2024). The economic impact is substantial, causing an estimated annual loss of $27.3 billion (Majeed and Muhammad, 2019). Additionally, agricultural land globally suffers from nutrient deficiencies, impacting crop yields and food security. Key deficiencies include nitrogen, phosphorus, and potassium, with micronutrients like zinc and iron also commonly lacking. As per recent reports, 30% of the world’s soils are suffering from iron deficiency, directly impacting crop yields and demanding environmentally friendly methods to restore productivity (Mahender et al., 2019). Critically, soil salinity and iron deficiency are closely linked, as high pH and ionic strength reduce iron availability, while Na^+^-induced stress impairs root function and iron uptake, causing functional deficiency even in iron-rich soils. These constraints threaten food security and emphasize the need for improved nutrient management. Biofertilizers offer a sustainable solution by using living microorganisms to improve soil health and crop yield. They can increase yields by 10–40% while reducing synthetic fertilizer use by up to 25% (Ammar et al., 2023). Despite the development of biofertilizers from terrestrial microbes, limitations such as low efficiency, poor adaptability, and weak stress tolerance persist. This has shifted attention toward aquatic microbes, which offer better adaptability and resilience under stress.

Aquatic ecosystems offer a rich and diverse source of biofertilizers that can significantly enhance agricultural productivity and offer a sustainable and eco-friendly alternative to traditional chemical fertilizers (Kumar et al., 2021). Biofertilizers derived from aquatic ecosystems include extracts of seaweed, phytoplankton, microalgae, bacteria such as *Pantoea agglomerans*, and sun-dried marine detritus (Pajuelo et al., 2021). This investigation used the freshwater magnetotactic bacterium *Magnetospirillum gryphiswaldense* MSR-1 as a biofertilizer for tomato and paddy cultivation. MSR-1 was first isolated from the sediment of the Ryck river near Greifswald, Germany, by Schüler and was described by Schleifer et al. (1991). These bacteria are gram-negative, exhibit high motility, and possess a distinctive spiral shape, typically measuring 2–3 μm in length and magnetosomes about 35–120 nm in diameter (Katzmann et al., 2010; Tay et al., 2018). MSR-1 is microaerophilic, preferring environments with low oxygen levels (approximately 5–10 ppm relative dissolved oxygen), though it is also capable of growing under aerobic conditions (Jajan et al., 2019). MSR-1 possesses a 4.37 Mb genome with a high GC content of 63.28%, including 4,261 coding sequences. A significant genomic feature is the magnetosome island (MAI), comprising several gene clusters (*mms6, mamFDC, mamAB*, and *mamXY*, for a total of 27 genes; 23.6 kb) that encode the majority of magnetosome proteins The G + C percentage of the MAI is 60.99%, and 33 genes were annotated as transposases, implying that the MAI is instable (Schübbe et al., 2009; Komeili, 2012). Further, transcriptome analysis of MSR-1 under varying oxygen levels showed that magnetosome genes (*mam* and *mms*) are not oxygen-regulated, while microaerobic conditions activate denitrification and modify metabolism to support magnetosome synthesis (Wang et al., 2016). In addition to this, the MSR-1 genome also contains a range of agriculturally advantageous genes, including the *nif* gene for N_2_ fixation (Jiang, 2002; Pang et al., 2024). Moreover, the ferric uptake regulator (*fur*) gene and ferrous iron transport protein B (*feoB*) genes also influence magnetosome formation by regulating genes involved in iron absorption and transportation, as well as *pstB* and *phoU* for regulating and absorbing phosphate (Wang et al., 2014). MSR-1 also synthesizes intracellular magnetic particles known as magnetosomes, composed of magnetite (Fe_3_O_4_), which enhance iron bioavailability to plants (Uebe et al., 2018). MSR-1 also enhances plant stress tolerance through the expression of antioxidant genes such as catalase (*katE*) and superoxide dismutase (*sodB*), and the production of osmolytes such as proline, trehalose, and glycine betaine. Additionally, the presence of *IrrA* and *IrrB* also provides oxidative stress tolerance, aiding MSR-1 survival under fluctuating oxygen levels (Wang et al., 2017). Collectively, the genetic traits of MSR-1 support enhanced plant growth, higher yield, and sustainable, eco-friendly farming. As microaerophilic anaerobes, they inhabit the oxic–anoxic transition zone (OATZ) in soil, where they form magnetite crystals and contribute to nitrogen fixation, phytohormone production, organic matter breakdown, and nutrient release, thereby promoting plant development (Lefèvre and Bazylinski, 2013; Li et al., 2021). Moreover, MSR-1 is an eco-friendly bacterium that supports rhizosphere bacteria, enhancing overall plant development. Furthermore, MSR-1 produces biocompatible magnetosomes, enabling it to act as a PGPR that enhances yield and supplies iron under deficiency conditions (Shirsat and Suthindhiran, 2025). Although MSR-1 and other magnetotactic bacteria have been widely studied for bioremediation, imaging, and medical applications, their agricultural potential remains largely unexplored, highlighting the need to investigate their role as plant growth-promoting agents (Jacob and Suthindhiran, 2016). Consequently, this study develops a *Magnetospirillum* liquid biofertilizer (MLB) using MSR-1 to enhance growth, nutrient uptake, and stress tolerance in paddy (Trichy-5) and tomato (Shivam). By evaluating different MLB doses and assessing plant biochemistry, antioxidants, yield, and elemental uptake, the work fills a key gap in MTB-based agriculture and proposes a scalable, eco-friendly alternative to chemical fertilizers.

## 2 Materials and methods

### 2.1 Culturing of MSR-1 strain

*Magnetospirillum gryphiswaldense* (MSR-1) strain was obtained from the Deutsche Sammlung von Mikroorganismen und Zellkulturen (DSMZ), Germany, and cultured using the anaerobic Hungate method in Hungate tubes and serum bottles (Hungate, 1969). MSR-1 was grown in a modified ATCC-1653 *Magnetospirillum* Growth Medium (MSGM), formulated with KH_2_PO_4_, NaNO_3_, organic acids, and sodium acetate, and enriched with Wolfe’s mineral and vitamin supplements, ferric quinate, and the redox indicator resazurin. The medium was adjusted to pH 6.7, with vitamin and ferric components filter-sterilized before being added. Once the MSGM was prepared and dispensed into Hungate tubes or serum bottles (100 mL per bottle), the medium was rendered microaerophilic by sparging with 92% nitrogen gas for 2 min. The bottles were then immediately sealed with butyl rubber stoppers, screw-capped, and autoclaved at 121 °C to ensure sterility (Menghini et al., 2021). After cooling, inoculation with the MSR-1 culture was done aseptically using sterile syringes through the rubber septum. The inoculated cultures were incubated at 28 °C to 30 °C for 72 h. To assess bacterial growth and the presence of magnetotactic behavior, the serum bottles were placed on a magnetic stirrer, and light scattering was evaluated manually under illumination, a method adapted from (Lefèvre et al., 2009) to confirm the presence of MTB.

### 2.2 Microscopic examination of MSR-1 using scanning electron microscopy (SEM)

The external morphology of MSR-1 was examined using SEM; ZEISS EVO18, Germany, following the protocol outlined by (Ammar, 2017). Briefly, bacterial cells were harvested by centrifugation at 7000 rpm for 10 min using a refrigerated centrifuge (Lark & LIHRC16). The resulting pellet was washed with 0.1 M phosphate buffer and then fixed in 0.25% glutaraldehyde prepared in the same buffer (pH 7.0) and incubated overnight at 4 °C (Marie et al., 2014). After fixation, cells were rinsed 2–3 times with phosphate buffer to eliminate remaining fixative. Further, dehydration was performed using a graded ethanol series (30 to 100%), with each step lasting 10–15 min. Finally, the sample was incubated in 100% ethanol for 1 h to ensure complete dehydration. Once dried, the samples were sputter-coated with a thin layer of gold to improve surface conductivity. SEM imaging was conducted at an accelerating voltage of 5–15 kV using the ZEISS EVO18 microscope (Czerwińska-Główka and Krukiewicz, 2021).

### 2.3 Magnetospirillum based liquid biofertilizer (MLB) preparation

MSR-1 was cultured in MSGM and incubated at 28–30 °C for 72 h. The bacterial culture was harvested by centrifugation at 7,000 rpm for 10 min at 4 °C. The resulting cell pellet was resuspended in sterile distilled water to a final volume of 50 mL. The cell suspension’s turbidity was adjusted to match the 0.5 McFarland standard (OD600 ≈ 0.08–0.1), corresponding to approximately 1.5 × 10^8^ cells/mL, using a spectrophotometer (Zapata and Ramirez-Arcos, 2015; Mahesh et al., 2025). This standardized suspension was used as the stock culture for subsequent applications. Serial dilutions of the stock culture were prepared in sterile distilled water to obtain concentrations of 100, 80, 60, 40, and 20% (v/v) (FNCA, F. for N.C. in A, 2014). Each dilution was thoroughly mixed and stored at 4 °C for short-term use. Fresh preparations were made for each application to ensure viability (Himanshi and Yadav, 2022; Fatimah Hidayat et al., 2025).

### 2.4 Population growth studies of MSR-1 in soil

To assess MSR-1 survivability in soil, 5 mL of actively growing culture (~10^8^ cells/mL) was added in 100 g of autoclaved soil (1:1 ratio of red soil: garden soil) and incubated at 28–30 °C for 14 days. After incubation, 5 g of MSR-1-inoculated soil was suspended in 35 mL of sterile distilled water, vortexed, and allowed to settle. From this, 1 mL of the supernatant was transferred into fresh MSGM broth and incubated in Hungate tubes at 28–30 °C for 72 h. From these tubes, 100 μL was added onto MSGM semi-solid agar and maintained under microaerophilic conditions (92% N₂ sparging for 2 min) to confirm MSR-1 survival through characteristic band formation (Le Nagard et al., 2018). Growth dynamics in soil were further examined by inoculating 100 μL of the soil suspension, previously grown in Hungate tubes, into fresh MSGM broth. Cultures were incubated at 28–30 °C, and OD_600_ was recorded every 2 days for 14 days using a UV–visible spectrophotometer (Fuduche et al., 2019). Cell concentration was estimated semi-quantitatively using a McFarland standard curve, and growth curves were generated to determine lag, log, stationary, and decline phases (Zhang et al., 2011; Berny et al., 2020). The protocol was adapted from standard soil survivability assays for PGPR (Lee et al., 2021).

### 2.5 Root exudates analysis

Tomato (*Solanum lycopersicum*) and paddy (*Oryza sativa*) seeds were surface-sterilized in 1% sodium hypochlorite for 5 min and rinsed thoroughly with sterile distilled water. Seeds were germinated on petri dishes under controlled conditions (25 ± 2 °C; 16/8 h light/dark). Seedlings were then transplanted into pots containing sterilized soil and grown at 25 ± 2 °C, 60% RH, and 200 μmol/m^2^/s light intensity (Berqdar et al., 2025). Plants were irrigated as needed with sterile, carbon-free Hoagland’s solution to reduce background organic carbon (Hoagland, 1950). After 5 weeks, plants were uprooted, and roots were washed with sterile water. Root surfaces were sterilized again using 1% sodium hypochlorite for 2–3 min and rinsed thoroughly. Sterilized roots were placed in sterile glass beakers containing 100 mL carbon-free Hoagland’s solution (pH 6.5), gently aerated, and maintained at 25 °C with 200 μmol/m^2^/s light. The transfer of roots from soil to carbon-free Hoagland’s solution may introduce stress-related metabolite leakage in addition to physiological root exudates. To minimize this effect, plants were allowed a 2 h recovery period in fresh Hoagland’s solution prior to exudate collection, and only the subsequent collection fraction was analyzed. Roots were incubated for 4 h to allow exudate release. The exudate-containing solution was then collected and passed through a 0.22 μm filter to remove debris and microorganisms. The filtrate was evaporated in a hot-air oven to obtain concentrated root exudates, which were analyzed using HR-LCMS. Identified metabolites were matched using the NIST library, KEGG compound database, Metabolomics Workbench, and MassBank (Salem et al., 2022; Yan et al., 2024).

### 2.6 SEM analysis of the association between MSR-1 and selected plants

Plants were grown in sterile soil (1:1 ratio of red soil: garden soil) by autoclaving soil at 121 °C for 20 min, repeated twice at 24-h intervals, and confirmed microbe-free by plating (Hu et al., 2020). Seeds were surface-sterilized in 1% sodium hypochlorite for 5–10 min (Bošnjak Mihovilović et al., 2024). Sterilized seeds were germinated and transplanted into pots. MLB was applied either to the soil near roots or as a foliar spray every 10 days. Control plants received sterile water. After 30 days, plants were harvested for SEM analysis. Roots and leaves samples were fixed in 2.5% glutaraldehyde and 37% formaldehyde in 0.1 M phosphate buffer (pH 7.2) for 2–4 h, then rinsed three times in the same buffer. Dehydration followed a graded ethanol series (30–100%). Fully dehydrated samples were air-dried, mounted on SEM stubs with conductive adhesive, and sputter-coated with gold or Au/Pd. Prepared samples were imaged under SEM at suitable accelerating voltages and magnifications to observe MSR-1 morphology and distribution on root and leaf surfaces (Baldotto and Olivares, 2008; Singh et al., 2021; Baptista et al., 2022).

### 2.7 Study area and experimental conditions

The pot experiment was conducted at the greenhouse at Vellore Institute of Technology (VIT, Vellore) situated at 12.967866° N and 79.160514° E. The environmental conditions in the study area were characterized by an average temperature of 27.1 °C, with a fluctuation range of ±2.5 °C, and a relative humidity level around 72%, with a variation of ± 10%. The plants were exposed to a photoperiod consisting of 10–12 h of sunlight and 12–14 h of darkness.

### 2.8 Selection of seeds

For this study, crop varieties were selected: Tomato (Shivam) and Paddy (Trichy-5) representing vegetable, and food grain crops, respectively. The choice of these plants was based on several important parameters such as their economic significance, widespread cultivation by farmers, sensitivity to soil nutrient imbalances such as iron deficiency and salinity, and relatively short maturity periods (Tomato: 85–90 days; Paddy: 110–115 days). All the seeds varieties were obtained from the Tamil Nadu Agricultural University (TNAU), Coimbatore, as they were adapted to the climate of the study region.

### 2.9 MLB application as a nitrogen and iron fertilizer

A pot culture experiment was conducted using tomato (Shivam) and paddy (Trichy 5) to evaluate the efficacy of MLB under iron deficiency and salinity stress. Iron-deficient soil was collected from fields showing chlorosis and analysed for available iron using a standard soil testing kit (Bai et al., 2018). Soil properties before and after treatment were assessed at the National Agro Foundation. For salinity stress, tomato pots were irrigated with 0.15% NaCl (2.5 dS/m) and paddy pots with 0.35% NaCl (6 dS/m). Soil pH and EC were monitored with a digital meter to confirm stress levels (Ali et al., 2023). EC was maintained at 2.5 dS/m for tomato and 6 dS/m for paddy to match crop tolerance; (Kalaiyarasi et al., 2019; Thakur et al., 2022). Clay soil was selected for paddy, while tomato was grown in a 1:1 mixture of red and garden soil (Dhanyalakshmi et al., 2013). For each treatment (Groups 1–8), 15 seeds were soaked in their respective solutions for 12 h before sowing. Treated seeds were germinated in pro trays and transplanted into sterilized pots (30 × 20 cm) after 20 days. Thinning was performed to maintain three plants per pot. Experiments followed a completely randomized design (CRD) with three replicates (Bandopadhyay, 2019). MLB was applied at 20, 40, 60, 80, and 100% concentrations, with 25 mL per pot every 10 days. Positive controls included 2% urea, *Azospirillum*, 6% Fe-EDTA, 0.2% gypsum, and *Pseudomonas fluorescens*; distilled water served as the negative control (Ay et al., 2022). Dosages followed TNAU and Fertilizer Control Order (1985) recommendations (FAI, 2019). Plants were maintained under greenhouse conditions and irrigated every 2–3 days. Growth, biochemical parameters, and yield were recorded on day 90 (tomato) and day 110 (paddy). Soil and plant samples were analysed pre- and post-treatment to assess MLB effects under iron-deficient and saline conditions. Data were expressed as mean ± standard error of mean (SEM), and one-way ANOVA (*p* ≤ 0.05) was performed using JMP Pro (Version 17). Supplementary Table S1 summarizes the treatments under normal, iron-deficient, and saline stress conditions.

### 2.10 Physical and biochemical paramters analysis

At maturity, plants were carefully uprooted, washed to remove soil, and assessed for growth parameters. Root and shoot lengths were measured, and fresh weight was recorded before drying samples at 50 °C for 48 h to determine dry biomass. For paddy, mature seeds were collected and weighed to estimate yield. In case of tomato, number and weight of berries (fruits) per plant were measured. After treatments, plant tissues were harvested and stored at −80 °C for biochemical analyses (Moore, 2012). Further, samples were cold-homogenized to prevent degradation, and pigment levels were quantified spectrophotometrically at 645, 663, and 470 nm for chlorophyll a, chlorophyll b, and carotenoids. Total chlorophyll was calculated from the combined chlorophyll a and b values (Lichtenthaler and Wellburn, 1983). The carbohydrate concentration was quantified using the phenol-sulfuric acid assay (DuBois et al., 1956), while the total phenolic compounds in the plant sample were measured employing the Folin–Ciocalteu method (Sánchez-Rangel et al., 2013). The protein content was determined through the Lowry protocol (Lowry et al., 1951), while amino acids were quantified using the Ninhydrin method (Moore and Stein, 1948). The superoxide dismutase (SOD) and catalase activity were measured calorimetrically at 450 nm and 570 nm, respectively, using assay kits (ab65354 for SOD and ab83464 for catalase, Abcam, Cambridge, UK) following the protocol provided by the manufacturer. Furthermore, the Kjeldahl technique was employed to estimate the nitrogen content of the plant samples (Bradstreet, 1954). Lastly, ICP-OES analysis was conducted to measure the iron and sodium levels in the plant sample filtrate (Ngigi and Muraguri, 2019).

### 2.11 Soil analysis

Soil samples were collected from the VIT agricultural field to assess the influence of MLB under normal conditions without stress. Further, iron-deficient soil was gathered from a field where plants displayed iron deficiency symptoms, to investigate the impact of MLB under iron-deficient conditions (Sahrawat, 2016; Bai et al., 2018). Furthermore, to investigate the impact of MLB on plant growth enhancement under saline conditions, the soil was salinized using a sodium chloride solution until the electrical conductivity of the soil reached a maximum of 2.5 dS/m (Tomato) and 6 dS/m (Paddy) (Mondal and Borromeo, 2016; Kakar et al., 2019). Soil samples were collected from a depth of 0–15 cm, and then dried in a hot air oven for 6 h. The samples were passed through a 2 mm sieve and grounded uniformly to minimize particle size effects. Further, the total nitrogen content was assessed using the Kjeldahl method (Bradstreet, 1954), while the iron content in the soil samples was estimated through the DTPA soil test (Lindsay and Norvell, 1978). Thereafter, the soil nutrient analysis was conducted in accordance with the guidelines outlined in the “Guide to Laboratory Establishment for Plant Nutrient Analysis” (Motsara and Roy, 2008).

## 3 Results

### 3.1 Morphological assessment of MSR-1

**Figure 1:**
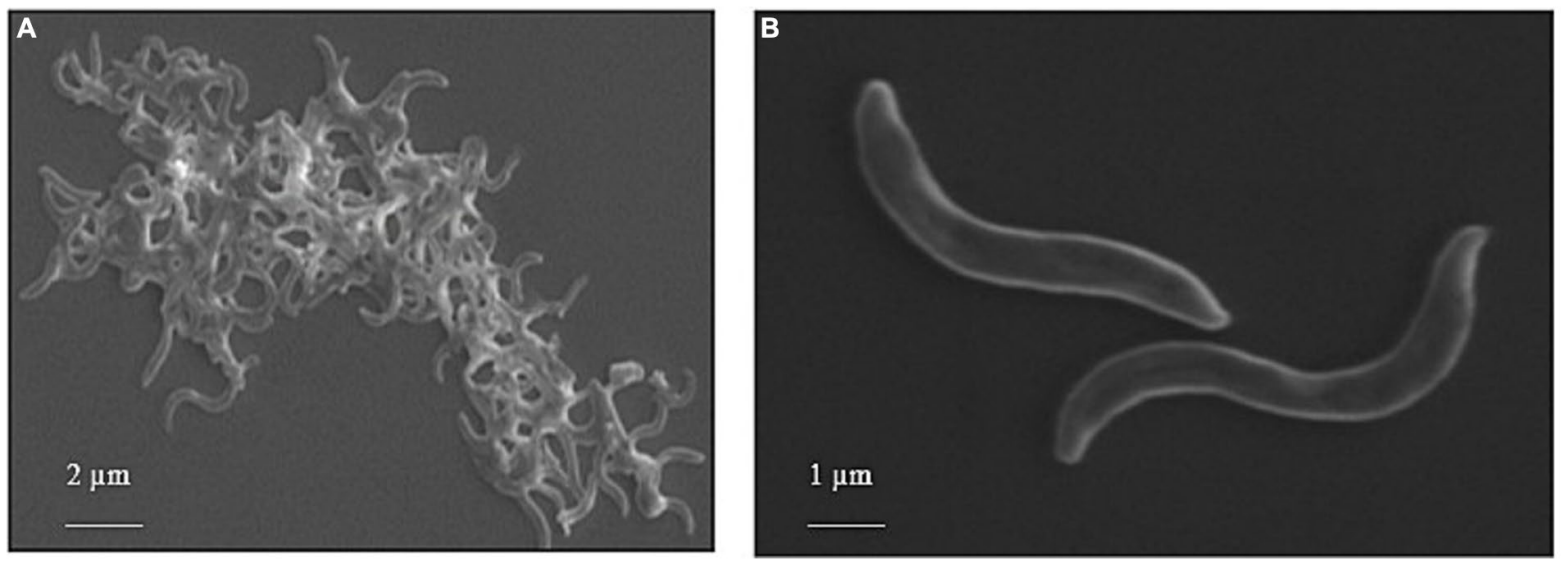
Morphology and structure of MSR-1. **(A)** Cluster of MSR-1 cells and **(B)** MSR-1 strain showing spiral-shaped structure.

The morphological features of MSR-1 were examined using SEM to confirm cell shape and structural integrity. The SEM images showed numerous uniformly distributed, spiral-shaped cells (Figure 1A), indicating healthy morphology under the culture conditions. A close-up image of two individual cells (Figure 1B) clearly displays the typical spiral form of *Magnetospirillum*, measuring about 1 μm in length with a smooth outer surface. These observations confirm the identity of the organism as MSR-1 and highlight its consistent morphology in laboratory conditions.

### 3.2 Assessment of growth studies of MSR-1 in soil

**Figure 2:**
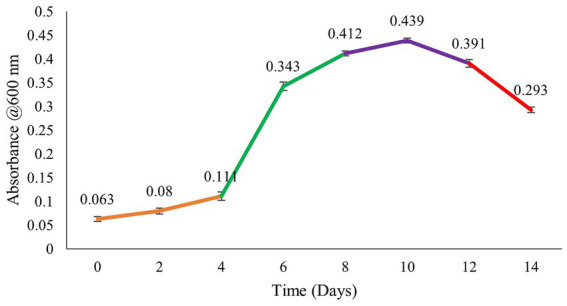
MSR-1 growth curve observed for a period of 14 days shows different phases of growth.

The soil preculture introduced into semi-solid agar showed microaerophilic growth rings near the oxic-anoxic boundary, confirming the presence of MSR-1 (Supplementary Figure S1). In tube ‘b.’, a distinct band of bacterial growth is observed near the top of the semi-solid agar suggesting that the bacteria are thriving in microaerobic conditions This indicates the presence of magnetotactic bacteria. Tube ‘a.’ serves as a blank, negative control indicating no visible band of growth, indicating the absence of magnetotactic bacteria. Over a 14-day period, MSR-1 demonstrated consistent growth in soil for 10 days before entering a death phase after day 12. The growth curve of MSR-1 indicated an initial OD of 0.063 at 600 nm, reaching a peak OD of 0.439 on day 10 (Figure 2).

### 3.3 Investigation of interaction between MSR-1 and tomato, and paddy

#### 3.3.1 SEM analysis between MSR-1 and the roots of plants

**Figure 3:**
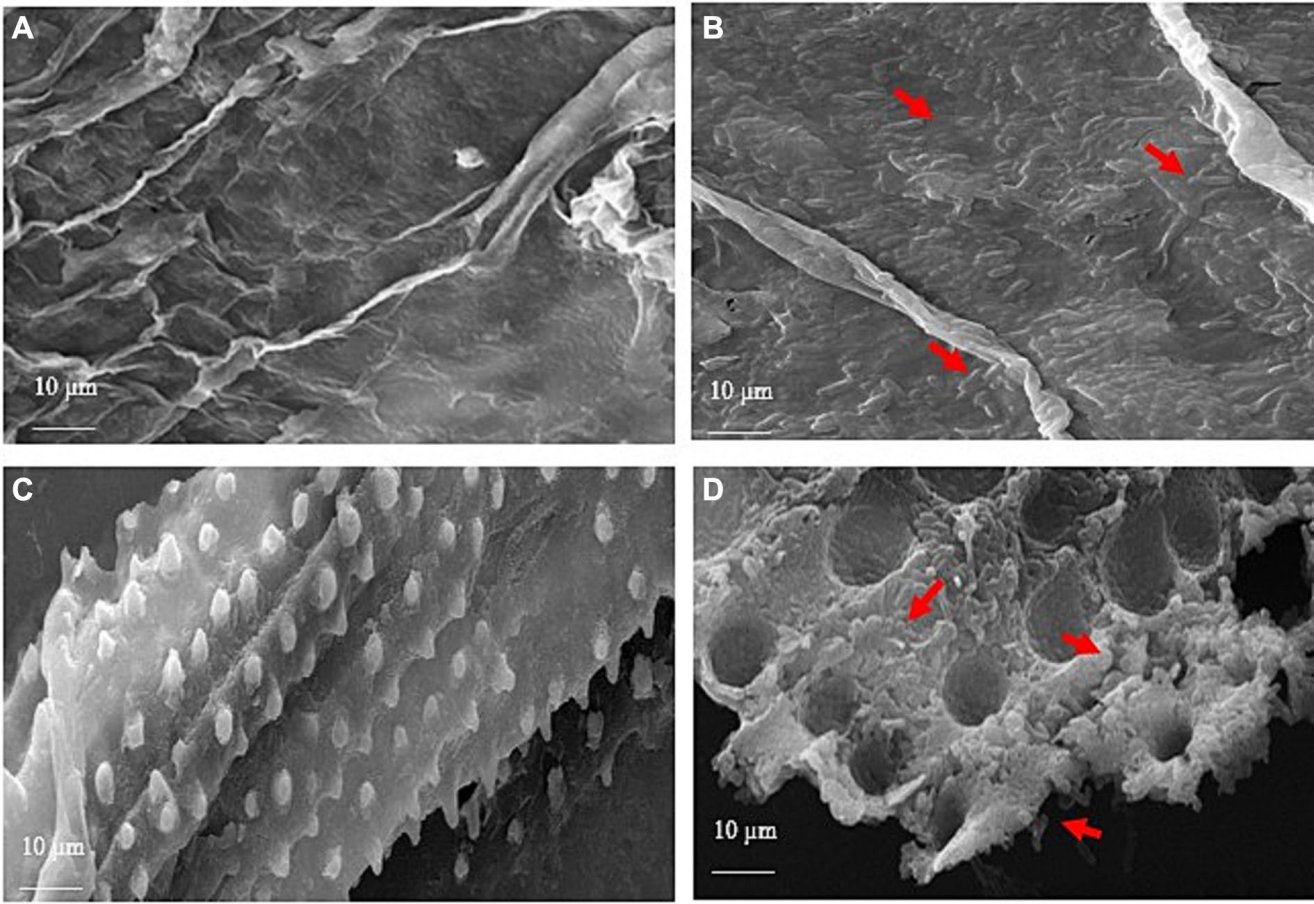
SEM analysis between MSR-1 and the roots of plants. **(A)** Root of tomato (Control), **(B)** Root of tomato (Treated with MSR-1), **(C)** Root of paddy (Control), and **(D)** Root of paddy (Treated with MSR-1).

The root colonization potential of MSR-1 was examined using SEM in tomato and paddy (Figure 3). Control roots displayed normal morphology for example, tomato roots showed fibrous structure with pronounced longitudinal ridges, while paddy roots had compact epidermal layers. After MSR-1 inoculation, in tomato, MSR-1 cells accumulated within surface folds and ridges, forming visible aggregates. In paddy, the bacteria adhered mainly along epidermal grooves and furrows, aligning in elongated patterns across the surface. Overall, SEM analysis confirmed effective MSR-1 adherence and colonization without causing structural damage, demonstrating its compatibility with diverse root systems and supporting its potential as a biofertilizer.

#### 3.3.2 SEM analysis between MSR-1 and the leaves of the plants

**Figure 4:**
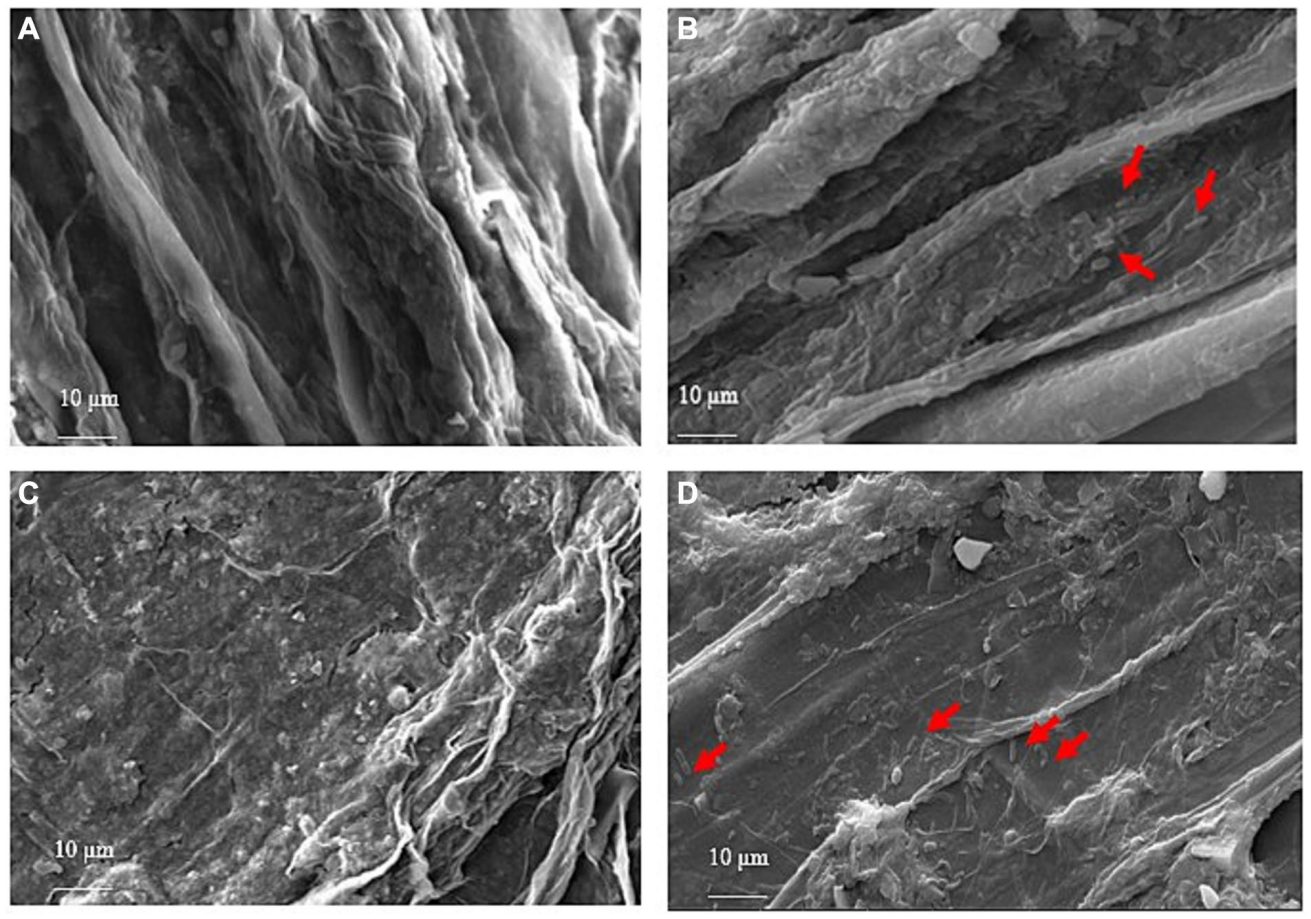
SEM analysis between MSR-1 and the leaves of plants. **(A)** Tomato leaf (Control), **(B)** Tomato leaf (Treated with MSR-1), **(C)** Paddy leaf (Control), and **(D)** Paddy leaf (Treated with MSR-1).

Comparative SEM micrographs show the leaf surface morphology of tomato and paddy under control conditions and after association with MSR-1 (Figure 4). Control leaves display intact epidermal layers with smooth surfaces, while paddy leaves show well-defined trichomes. In MSR-1-treated samples, red arrows highlight clear microbial colonization. Tomato leaves exhibit localized attachment of MSR-1 at the marked sites without disrupting overall surface integrity. In paddy, MSR-1 is observed adhering to trichome bases and epidermal regions, with circular cavities indicative of site-specific biofilm formation. The leaf’s rough surface promotes bacterial adhesion and interaction. These micrographs confirm successful, non-destructive MSR-1 colonization in both species, demonstrating a compatible plant–microbe relationship.

### 3.4 Analysis of root exudates using HR-LCMS

#### 3.4.1 Root exudate profile of tomato

HR-LCMS profiling of tomato root exudates showed a diverse mixture of primary and secondary metabolites, including organic acids, amino acids, sugars, flavonoids, diterpenoids, sterols, and phospholipids (Table 1). The mass spectrum, plotted with m/z on the x-axis and relative ion intensity on the y-axis, displayed multiple distinct peaks representing various ionized compounds (Supplementary Figure S2), confirming the chemical complexity of tomato exudates. Metabolites ranged from low to high molecular weight. Among the low-m/z compounds, quinic acid was identified as a strong microbial chemoattractant, while tryptophan a key signaling amino acid was also detected. Quercetin, a flavonoid known for antioxidant activity and its role in attracting beneficial microbes, was present. Higher-m/z metabolites included gibberellin A7 (a diterpenoid hormone), stigmasterol (a phytosterol), and 4-methylsulfinylbutyl glucosinolate, all associated with plant growth regulation, defense, and microbial recruitment. Additional detected compounds such as chicoric acid and flavonol 3-O-*β*-D-glucosyl-(1 → 2)-β-D-glucoside act as chemoattractants involved in plant–microbe symbiosis. At the upper end of the spectrum, UDP-N-acetylglucosamine and phosphatidylethanolamine were observed. Overall, the metabolite profile demonstrates that tomato roots release a chemically rich blend of primary and secondary metabolites, many of which serve as chemoattractants. This highlights the active role of root exudates in shaping rhizosphere microbial communities and facilitating beneficial plant–microbe interactions.

**Table 1:** Representative metabolites identified from the root exudates of tomato along with their corresponding m/z values and chemical classifications.

Sr. No.	Compound name	m/z	Nature of compound	Property of compound	References
1	Quinic acid	192.09	Organic acid	Chemoattractant	Wen et al. (2022)
2	Tryptophan	205.021	Amino acid	Chemoattractant	Fagorzi et al. (2021)
3	Quercetin	302.27	Flavonoid	Chemoattractant	Gargallo-Garriga et al. (2018)
4	Gibberellin A7	330.304	Diterpenoid	High MW	Mannucci et al. (2022)
5	Stigmasterol	412.353	Sterol	High MW	Meena et al. (2017)
6	4-Methylsulfinylbutyl glucosinolate	437.168	Glucosinolate	Chemoattractant	Mannucci et al. (2022)
7	Chicoric acid	475.296	Organic acid	High MW	Ma et al. (2022)
8	Flavonol 3-O-beta-D-glucosyl-(1->2)-beta-D-glucoside	563.358	Flavonoid	Chemoattractant	Mannucci et al. (2022)
9	UDP-N-acetylglucosamine	607.39	Sugar derivative	High MW	Nadal et al. (2017)
10	Phosphatidylethanolamine	739.483	Phospholipid	High MW	Couvillion et al. (2024)

#### 3.4.2 Root exudate profile of paddy

Metabolomic profiling of paddy root exudates revealed a chemically diverse mixture of low- and high-molecular-weight compounds, including organic acids, phenolics, flavonoids, and various secondary metabolites (Table 2). The mass spectra showed distinct peaks, with the most prominent at m/z 389.15 corresponding to strigyl acetate, a strigolactone known for its signaling and chemoattractant roles in plant–microbe interactions. Another major peak at m/z 437.18 was identified as 4-methylsulfinylbutyl glucosinolate, a sulfur-rich defense compound linked to microbial attraction in the rhizosphere. Additional peaks represented organic acids, flavonoids, strigolactones, glucosinolates, and phenolic derivatives (Supplementary Figure S3). Organic acids such as citric acid, palmitic acid, and 2-hydroxy stearic acid were detected, indicating the importance of low-molecular-weight metabolites in nutrient solubilization, signaling, metabolic activity, and stress adaptation. Several phenolic and flavonoid compounds including 5-O-coumaroylquinic acid and 5,2′,3′-trihydroxy-3,6,7-trimethoxyflavone were also present and are known chemoattractants for rhizosphere microbes. Additional flavonoids such as formononetin 7-O-glucoside, 6-C-glucopyranosylcatechin, and malonylgenistin further demonstrate the metabolite richness of paddy exudates. Bergapten, a coumarin derivative commonly associated with plant–microbe communication, defense, and allelopathy, was also detected. Overall, the wide range of organic acids, flavonoids, phenolics, glucosinolates, and strigolactones highlights the chemical complexity of paddy root exudates. Many of these metabolites possess chemoattractant functions, suggesting that paddy roots actively influence rhizosphere nutrient dynamics, defense responses, and beneficial microbial recruitment.

**Table 2:** Identified metabolites from paddy root exudates, along with their m/z values and chemical classifications.

Sr. No	Compound name	m/z	Nature of compound	Property of compound	References
1	Citric acid	192.1	Organic acid	Low mol.wt	Camli-Saunders and Villouta (2025)
2	Bergapten	216.89	Secondary metabolite	Low mol.wt	Afzal et al. (2025)
3	Palmitic acid	256.79	Organic acid	Low mol.wt	Meena et al. (2017)
4	2-Hydroxy stearic acid	301.11	Organic acid	High MW	Couvillion et al. (2024)
5	5-O-coumaroylquinic acid	359.20	Phenolic acid derivative	Chemoattractant	Meena et al. (2017)
6	5,2′,3′-Trihydroxy-3,6,7-trimethoxyflavone	361.09	Flavonoids	Chemoattractant	Meena et al. (2017)
7	Strigyl acetate	389.15	Strigolactone	Chemoattractant	Xie (2016)
8	Formononetin 7-O-glucoside	431.27	Flavonoids	Chemoattractant	Leoni et al. (2021)
9	4-Methylsulfinylbutyl glucosinolate	437.1	Glucosinolate	Chemoattractant	Mannucci et al. (2022)
10	6-C-Glucopyranosylcatechin	453.15	Flavonoids	Chemoattractant	Mannucci et al. (2022)
11	Malonylgenistin	519.33	Flavonoids	Chemoattractant	Ahmad et al. (2021)

### 3.5 Analysis of physical growth parameters of tomato and paddy

#### 3.5.1 Tomato

**Figure 5:**
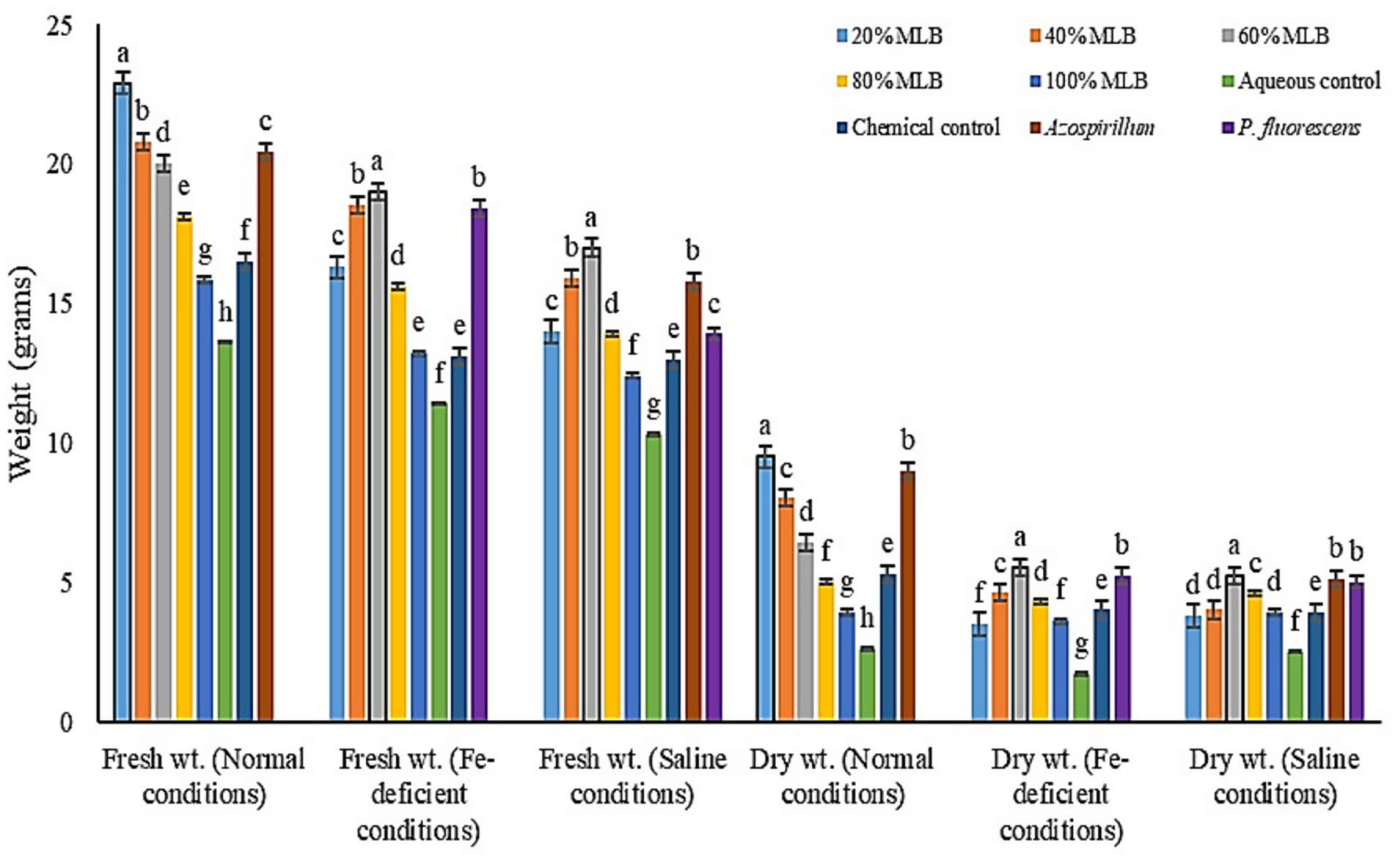
Effect of different treatments on root and shoot length of tomato under normal, Fe-deficient and saline conditions. Means ± SEM (*n* = 3); One-way ANOVA (*p* < 0.05).

Pot experiments in tomato showed that shoot and root lengths varied significantly across MLB concentrations under normal, Fe-deficient, and saline conditions (Supplementary Figure S4). Under normal conditions, 20% MLB produced the tallest shoots (73.5 cm), a 2.88-fold rise over the aqueous control (25.5 cm). Root length was also highest at 20% MLB (31.4 cm), a 1.9-fold increase over the control (16.4 cm), with *Azospirillum* showing similar values (69.43 cm and 30.53 cm). In Fe-deficient soil, 60% MLB resulted in maximum shoot length (60.3 cm), marking a 41% improvement over the aqueous control (42.6 cm) and 22% over the chemical control (49.5 cm). Root length also peaked at 60% MLB (28.6 cm), showing 43 and 12% increases compared to the aqueous and chemical controls, respectively, with *P. fluorescens* performing closely (57.2 and 27.9 cm). Under saline stress, 60% MLB again showed superior performance, producing the longest shoots (58.6 cm) with 44 and 10% gains over the aqueous (40.8 cm) and chemical controls (53.2 cm). Root length was highest at 60% MLB (26.8 cm), recording 40 and 12% increases over the respective controls. *Azospirillum* (57.3 and 26.6 cm) showed nearly equal performance, while *P. fluorescens* (56.6 and 25.9 cm) remained slightly lower but still better than the chemical control (Figure 5).

**Figure 6:**
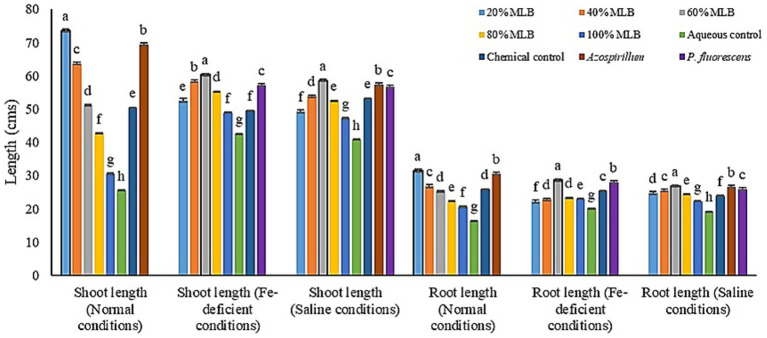
Impact of MLB and control treatments on the fresh and dry biomass of tomato under normal, iron-deficient, and saline conditions. Means ± SEM (*n* = 3); One-way ANOVA (*p* < 0.05).

In the study, it was found that fresh and dry weight varied significantly with different MLB concentrations and controls under normal, Fe-deficient, and saline conditions (Figure 6). Under normal conditions, 20% MLB (22.9 g fresh weight; 9.5 g dry weight) was the highest, followed by *Azospirillum* (20.4 g and 9.0 g respectively). The 20% MLB corresponded to a 68% increase in fresh weight and a 3.65-fold increase in dry weight over the aqueous control (13.6 g and 2.6 g, respectively). In Fe-deficient conditions, 60% MLB (19.0 g fresh weight; 5.5 g dry weight) was the maximum, showing a 67% increase in fresh weight and a 3.2-fold increase in dry weight over the aqueous control (11.4 g and 1.7 g respectively). Compared with the chemical control (13.1 g and 4.0 g), the increases were 45 and 37.5%, respectively whereas *P. fluorescens* (18.4 g and 5.2 g) closely matched 60% MLB. Under saline conditions, again 60% MLB (17.0 g fresh weight; 5.2 g dry weight) was superior, with a 65% rise in fresh weight and a 2.1-fold increase in dry weight compared to the aqueous control (10.3 g and 2.5 g).

**Figure 7:**
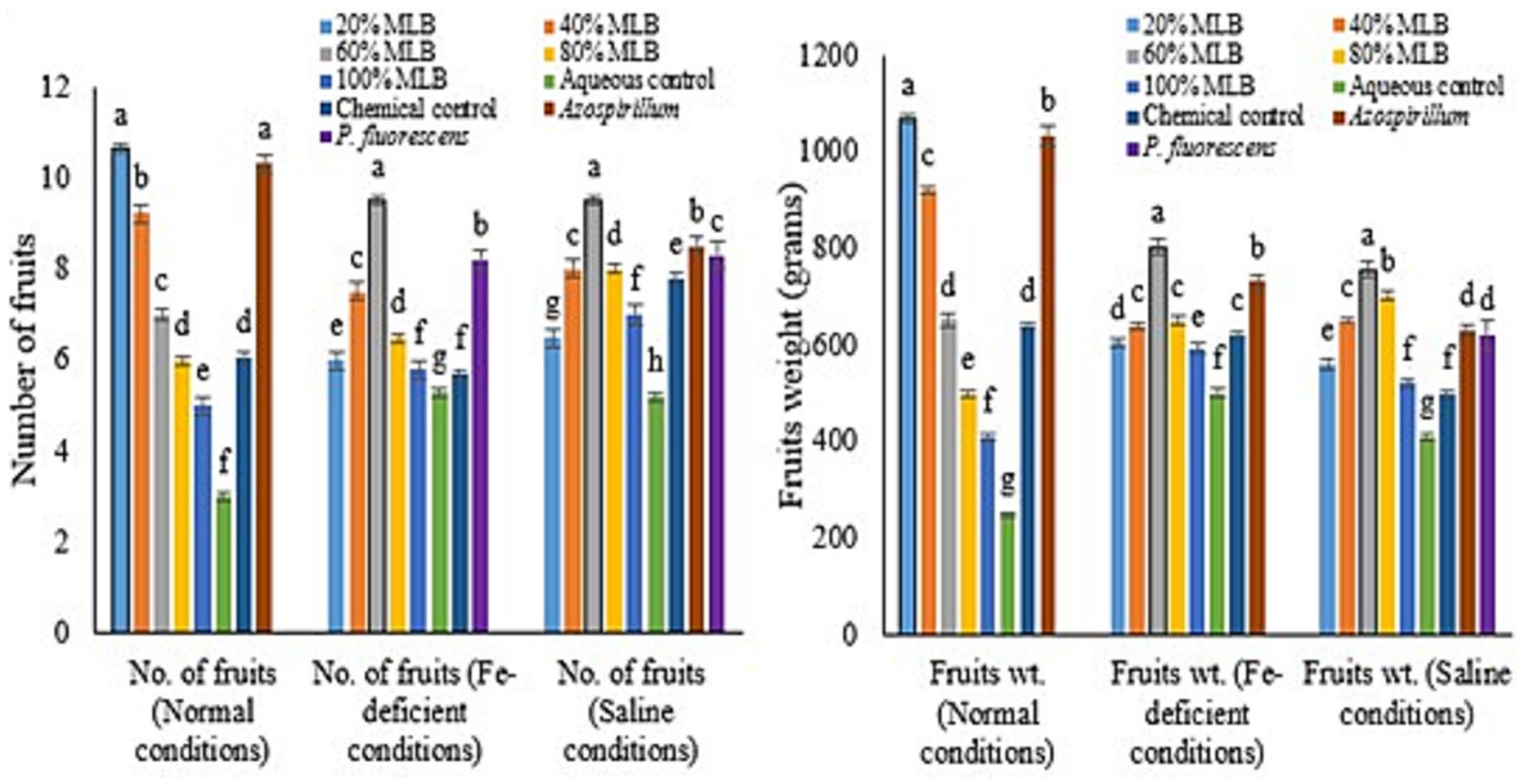
Comparison of the effects of MLB and controls on fruit weight and number of fruits per plant of tomato grown under optimal, Fe-deficient, and saline environments. Means ± SEM (*n* = 3); One-way ANOVA (*p* < 0.05).

Tomato fruit number varied noticeably across MLB concentrations under normal, Fe-deficient, and saline conditions. Under normal conditions, 20% MLB produced the highest fruit count (10.66), a 77.6% increase over the aqueous control (3 fruits). In Fe-deficient and saline soils, 60% MLB yielded the most fruits (9.5), outperforming both the aqueous (5.3 and 5.2) and chemical controls (5.7). Among other inoculants, *Azospirillum* showed comparable performance under normal conditions (10.33 fruits), while *P. fluorescens* performed moderately under Fe deficiency (8.2 fruits) and salinity (8.3 fruits). Fruit weight also differed significantly among treatments. Under normal conditions, 20% MLB produced the highest fruit weight (1066.95 g), a 4.3-fold increase over the aqueous control (250.1 g). In Fe-deficient soil, 60% MLB yielded the maximum fruit weight (802.66 g), giving a 60% increase over the aqueous control (500 g) and a 29% increase over the chemical control (620.89 g). Under saline conditions, 60% MLB again performed best (756 g), 1.8-fold higher than the aqueous control (410.8 g) and 1.5-fold higher than the chemical control (500.75 g). *Azospirillum* showed similar results under normal conditions (1033.55 g), while *P. fluorescens* performed moderately in Fe-deficient (730.9 g) and saline (620.97 g) soils. Overall, 20% MLB was most effective under normal conditions, whereas 60% MLB provided the greatest benefit under Fe deficiency and salinity (Figure 7).

**Figure 8:**
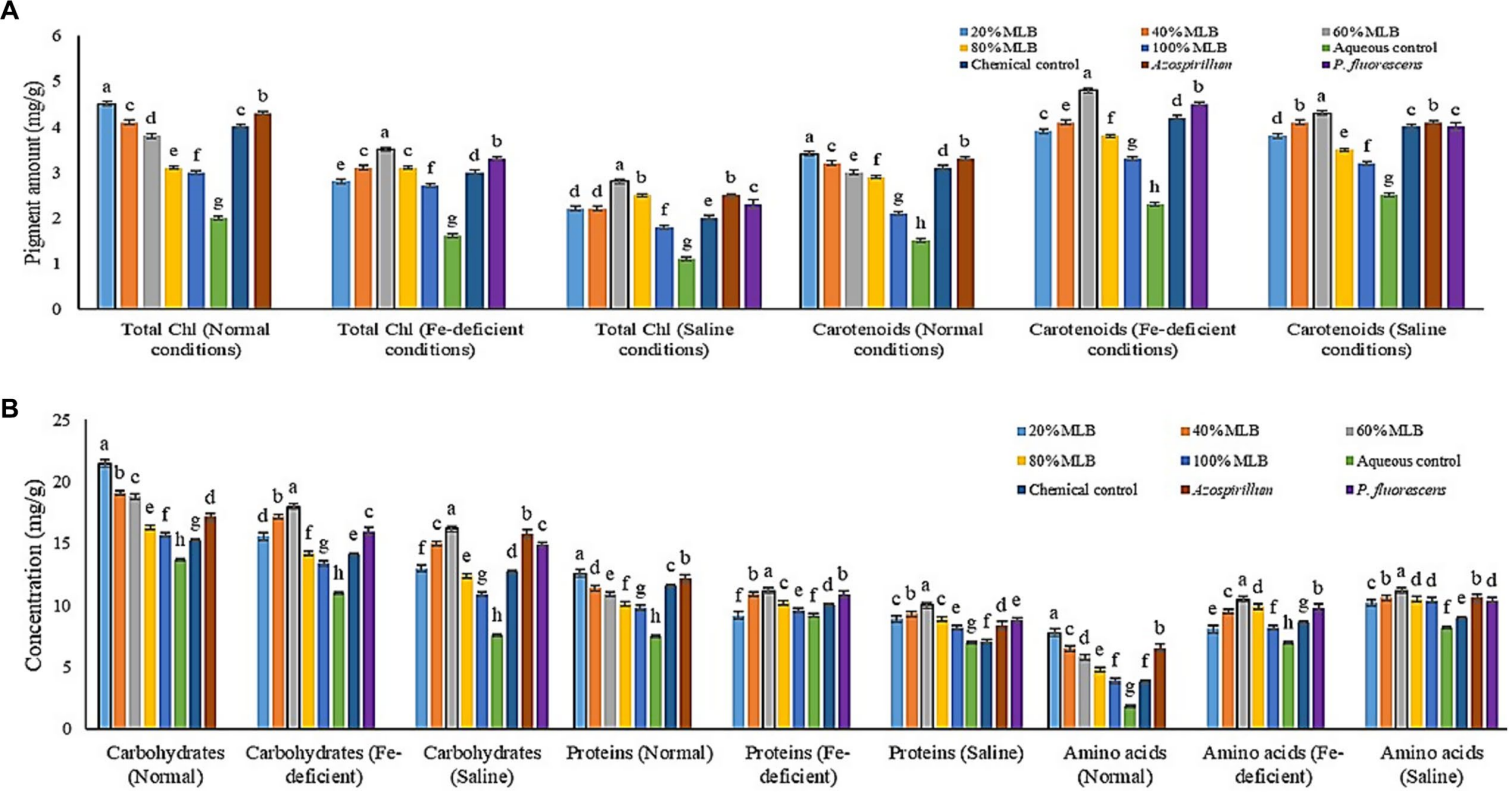
Assessment of effects of MLB and control treatments on tomato biochemical parameters across normal, iron-deficient, and salinity conditions. **(A)** Total chlorophyll and carotenoid content; **(B)** Carbohydrates, protein and amino acids content. Means ± SEM (*n* = 3); One-way ANOVA (*p* < 0.05).

**Figure 9:**
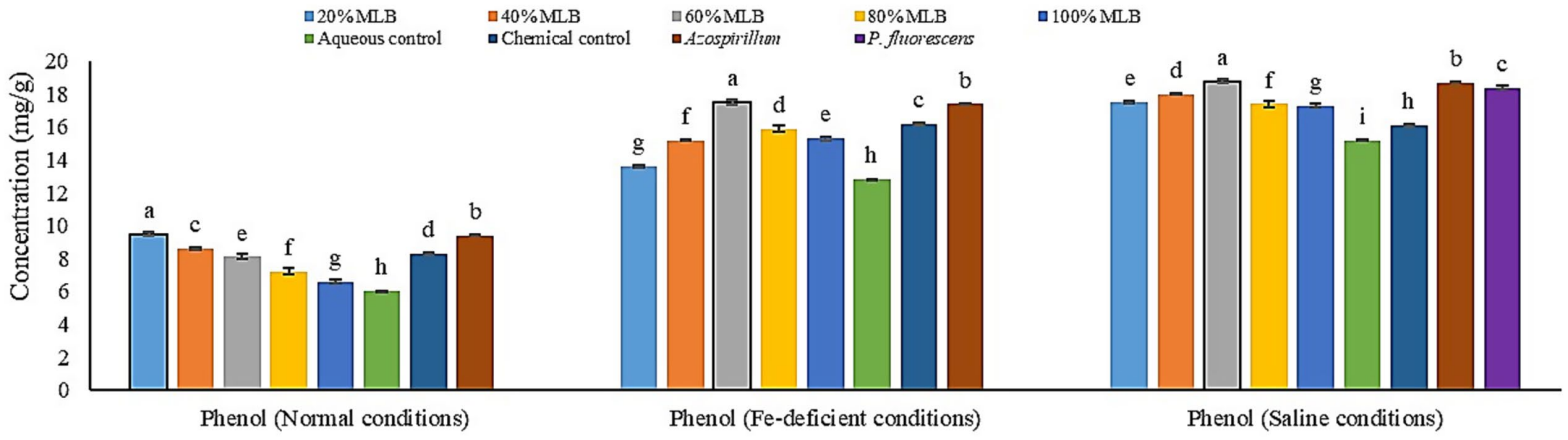
Impact of MLB and control treatments on the phenol content of tomato under normal, iron-deficient, and saline conditions. Means ± SEM (*n* = 3); One-way ANOVA (*p* < 0.05).

**Figure 10:**
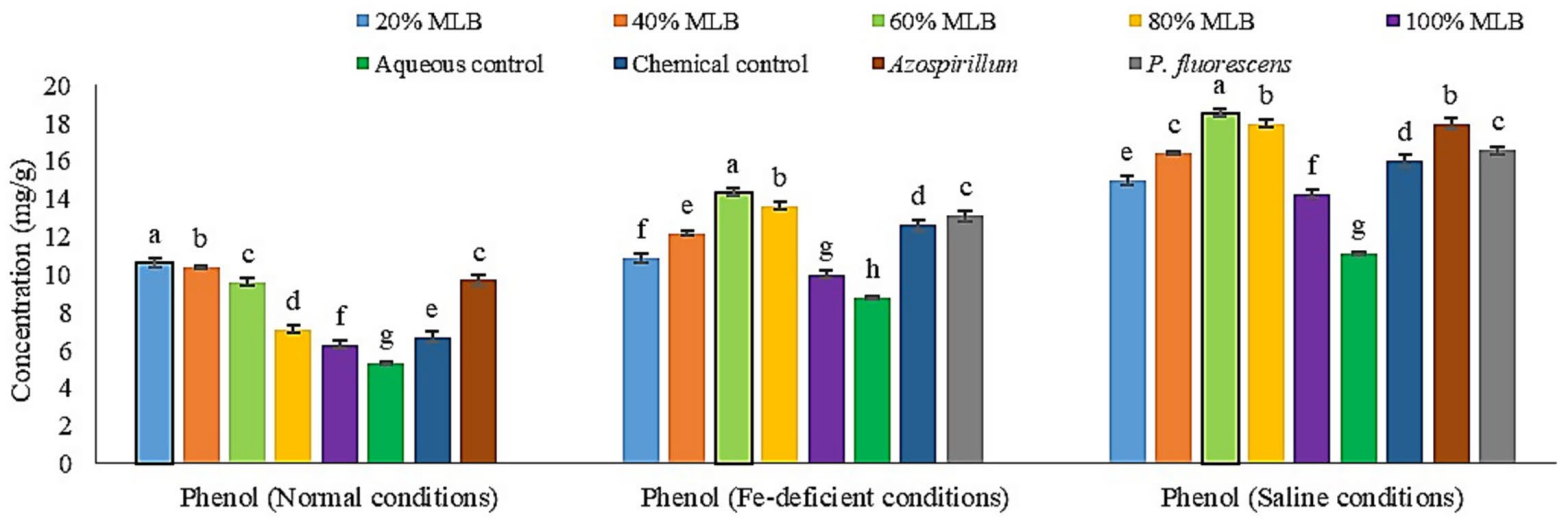
Assessment of the effects of MLB and controls on SOD and catalase of tomato under standard, iron-deficient, and saline growth conditions. Means ± SEM (*n* = 3); One-way ANOVA (*p* < 0.05).

MLB concentration markedly affected the biochemical traits of tomato (Sivam). Chlorophyll and carotenoid levels showed clear variation across treatments (Figure 8A). Under normal conditions, 20% MLB produced the highest chlorophyll content (4.5 mg/g), more than double the aqueous control (2.0 mg/g) and 12.5% higher than the chemical control (4.0 mg/g). In Fe-deficient and saline soils, 60% MLB recorded the highest chlorophyll values (3.5 and 2.8 mg/g), showing 2.2–2.5-fold increases over their respective aqueous controls. Carotenoid patterns followed the same trend, with 20% MLB performing best under normal conditions (3.4 mg/g), while 60% MLB maintained superior levels under Fe deficiency (4.8 mg/g) and salinity (4.3 mg/g), indicating better pigment stability during stress. Metabolic attributes carbohydrates, proteins, and amino acids also differed significantly (Figure 8B). Under normal conditions, 20% MLB recorded the highest carbohydrate content (21.5 mg/g), 1.6-fold higher than the aqueous control and 40.9% above the chemical control. Protein (12.6 mg/g) and amino acids (7.8 mg/g) were similarly elevated. Under Fe deficiency, 60% MLB maximized carbohydrates (18 mg/g), proteins (11.2 mg/g), and amino acids (10.5 mg/g). Salinity showed a similar pattern, with 60% MLB producing the highest values (16.2, 10, and 11.2 mg/g, respectively). *Azospirillum* consistently performed well under normal conditions, while *P. fluorescens* showed strong responses under stress, particularly for carbohydrate (14.9 mg/g) and amino acid (10.4 mg/g) levels. These results confirm that 20% MLB enhances metabolic pools under normal conditions, whereas 60% MLB is most effective during stress. Phenolic content also increased, especially under stress (Figure 9). Under normal conditions, 20% MLB recorded the highest phenols (9.5 mg/g), a 1.6-fold increase over the aqueous control. In Fe-deficient and saline soils, 60% MLB showed maximum phenol accumulation (17.5 and 18.8 mg/g), surpassing both controls and matching the performance of *Azospirillum* and *P. fluorescens*. Antioxidant enzymes SOD and CAT were strongly enhanced (Figure 10). Under normal conditions, 20% MLB recorded the highest SOD (57.2 units/mg) and CAT (10.0 units/mg), nearly double the aqueous control. Under Fe deficiency and salinity, 60% MLB again showed maximum SOD (72.5–75.0 units/mg) and CAT (15.0–15.5 units/mg) levels, exceeding both aqueous and chemical controls. Standard inoculants such as *Azospirillum* and *P. fluorescens* also displayed robust antioxidant activity under stress. The concurrent rise in phenolics and antioxidant enzymes suggests a coordinated enzymatic and non-enzymatic defense mechanism contributing to improved stress tolerance.

#### 3.5.2 Paddy

**Figure 11:**
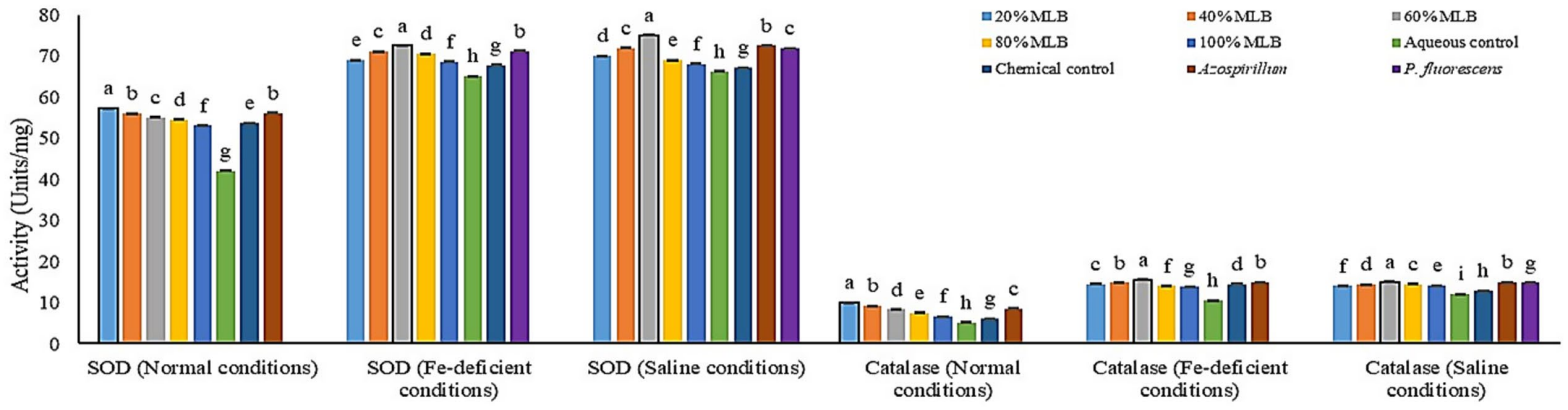
Effect of different treatments on root and shoot length of paddy under normal, Fe-deficient and saline conditions. Means ± SEM (*n* = 3); One-way ANOVA (*p* < 0.05).

The Figure 11 reveals variations in shoot and root dimensions under diverse treatments and environmental conditions. Under normal circumstances, the 20% MLB treatment yielded the greatest shoot (66 cm) and root length (15.13 cm), indicating its efficacy in promoting plant growth. Specifically, the 20% MLB treatment resulted in 60.98 and 219.68% increases in shoot and root length, respectively, compared to the control. In Fe-deficient conditions, the 60% MLB treatment demonstrated the highest shoot (55.83 cm) and root length (14.3 cm), with 36 and 150.88% increases over the control, suggesting that a higher MLB concentration is advantageous in iron-limited environments. Similarly, under saline conditions, the 60% MLB treatment again produced the maximum shoot length (52.5 cm) and root length (13.8 cm) with 36 and 42.27% increases compared to the control, underscoring its effectiveness in saline environments. The aqueous control groups consistently exhibited the lowest shoot and root lengths across all conditions, indicating that the other treatments significantly enhanced plant growth relative to water alone. While chemical control, *Azospirillum* and *P. fluorescens* also improved shoot and root lengths compared to the aqueous control, they were less effective than the optimal MLB concentrations (Supplementary Figure S5).

**Figure 12:**
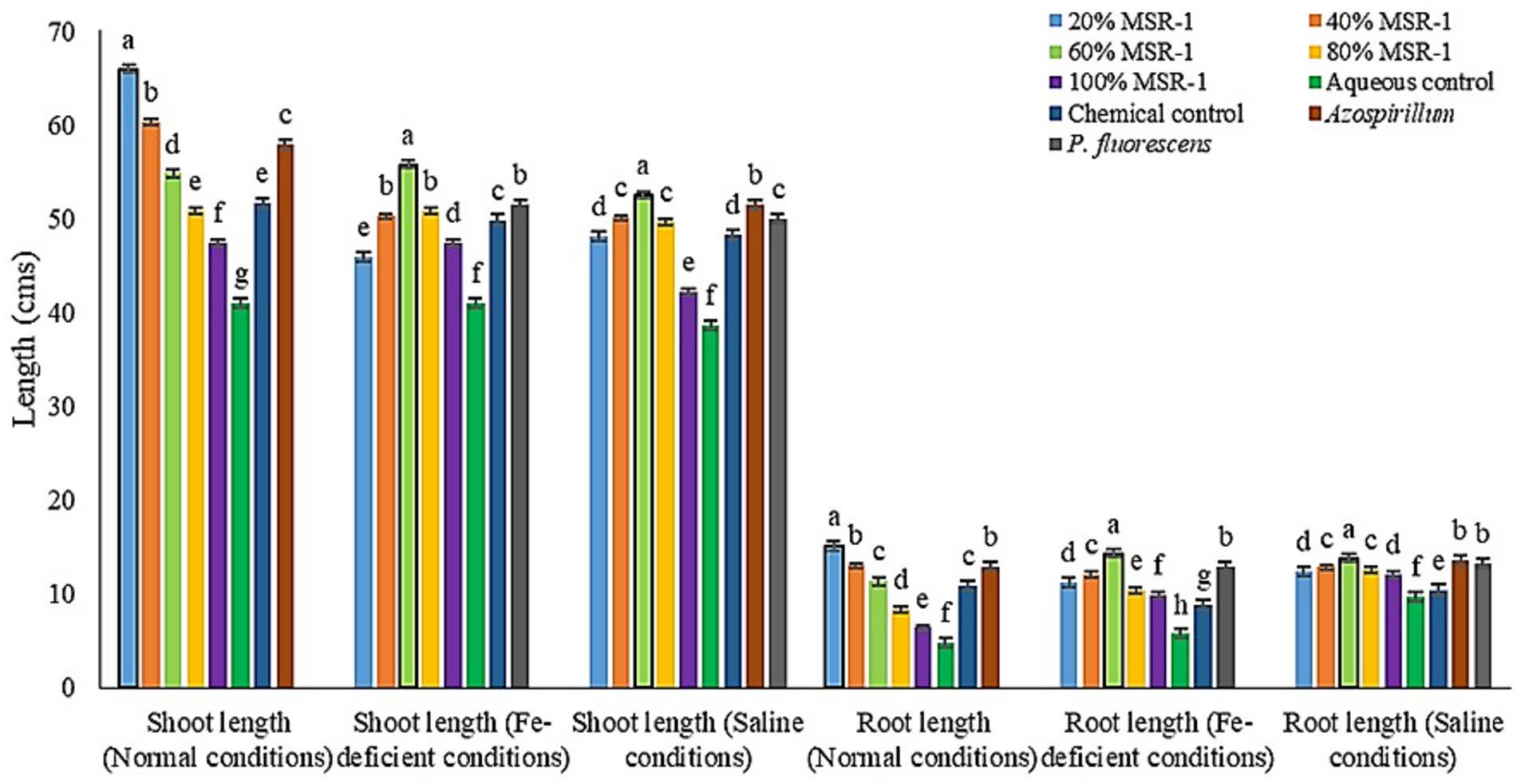
Effect of MLB and controls on fresh and dry weight of paddy under normal, Fe-deficient and saline conditions. Means ± SEM (*n* = 3); One-way ANOVA (*p* < 0.05).

**Figure 13:**
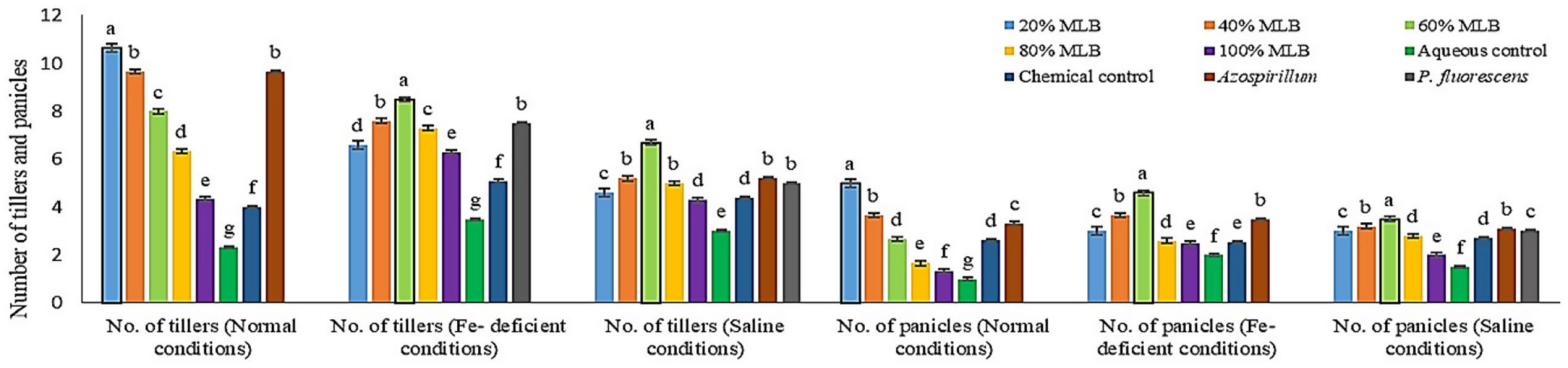
Comparison of effects of MLB and control on the number of tillers and panicles per plant of paddy grown under normal, Fe-deficient and saline conditions. Means ± SEM (*n* = 3); One-way ANOVA (*p* < 0.05).

Figure 12 illustrates the impact of different MLB concentrations, controls, and biofertilizers on fresh weight, dry weight, and seed weight under normal, Fe-deficient, and saline conditions. Under normal conditions, 20% MLB produced the highest fresh weight (50.16 g), while 100% MLB showed the lowest (40.5 g), a 19.3% reduction. In Fe-deficient soil, fresh weight peaked at 60% MLB (30.16 g), a 46% increase over the aqueous control (20.6 g). Under salinity, 60% MLB again performed best (29.8 g), nearly doubling the aqueous control (15.4 g). Dry weight followed similar patterns: 20% MLB was highest under normal conditions (20.1 g), whereas 60% MLB yielded the greatest dry weight under Fe deficiency (12 g) and salinity (12.21 g). The aqueous control showed 44.8, 41.7, and 51.7% lower dry weight than these optimal treatments. Seed weight was highest with 20% MLB (23.1 g) under normal conditions, an 85% improvement over the aqueous control (12.46 g). Under stress, 60% MLB, *Azospirillum*, and *P. fluorescens* consistently enhanced plant biomass and productivity, indicating strong stress-mitigation potential. Figure 13 further shows that 20% MLB produced the most tillers (10.67) and panicles (5.0) under normal conditions. However, 60% MLB resulted in the maximum tiller numbers under Fe-deficient (8.5) and saline environments, making it most effective for stress conditions.

**Figure 14:**
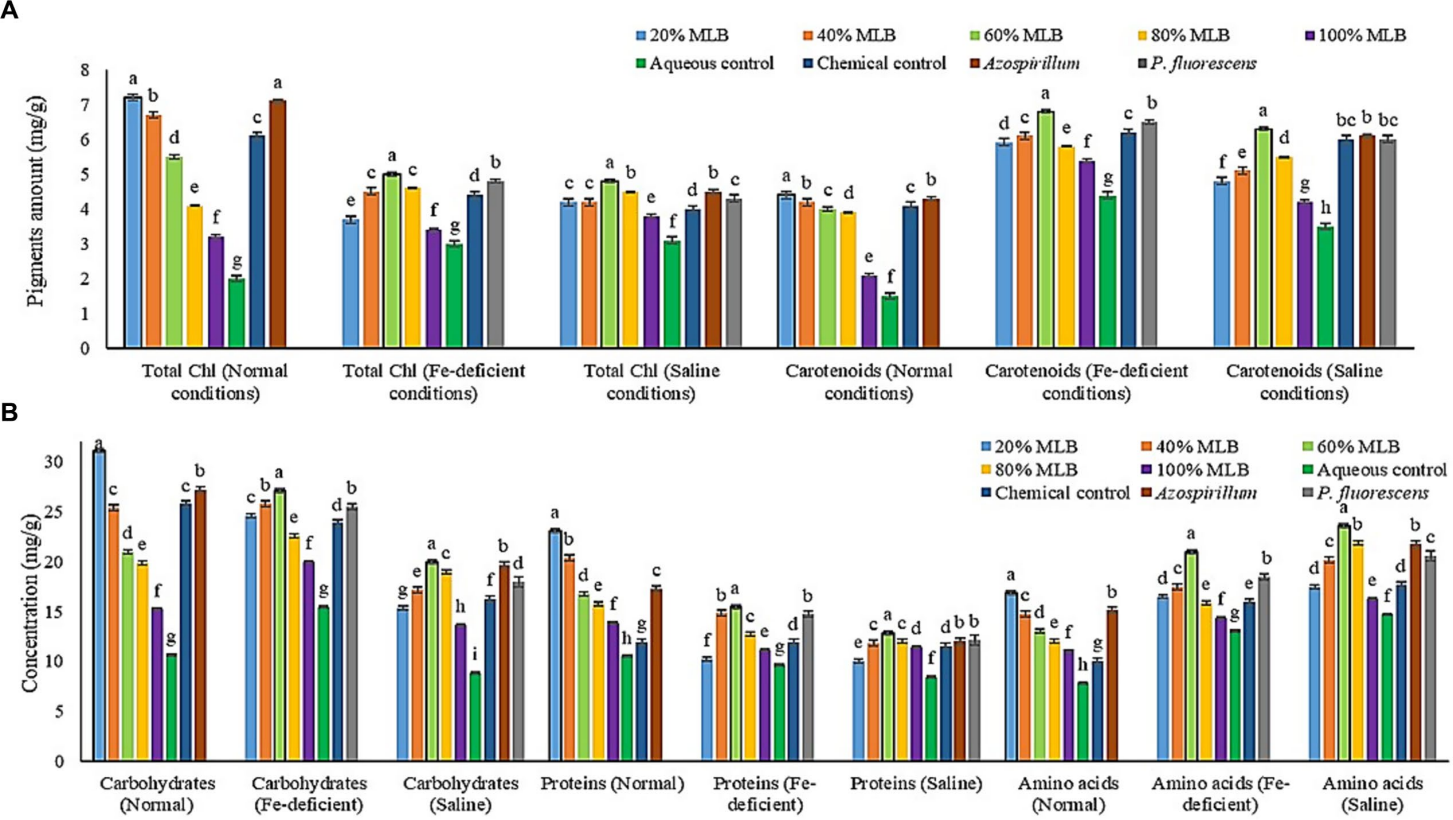
Assessment of effects of MLB and control treatments on paddy biochemical parameters across normal, iron-deficient, and salinity conditions. **(A)** Total chlorophyll and carotenoid content; **(B)** Carbohydrates, protein and amino acids content. Means ± SEM (*n* = 3); One-way ANOVA (*p* < 0.05).

**Figure 15:**
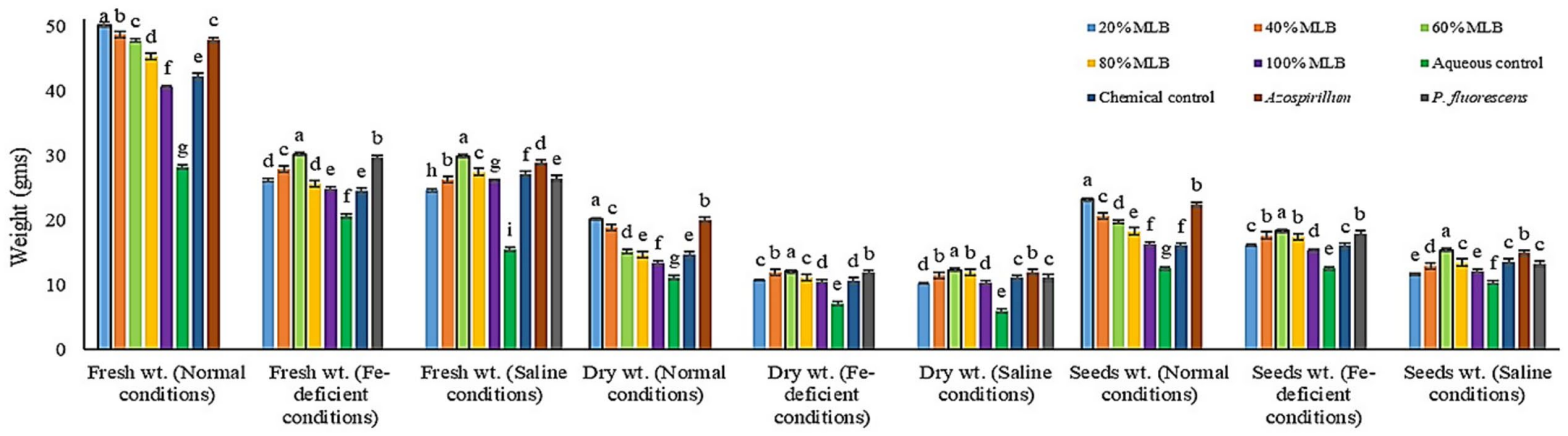
Impact of MLB and control treatments on the phenol content of paddy under normal, iron-deficient, and saline conditions. Means ± SEM (*n* = 3); One-way ANOVA (*p* < 0.05).

Figure 14A illustrates the influence of different MLB concentrations, controls, and biofertilizers on chlorophyll and carotenoid content under normal, Fe-deficient, and saline conditions. Under normal conditions, 20% MLB produced the highest chlorophyll (7.2 mg/g) and carotenoid levels (4.4 mg/g), followed by 40% MLB, while the aqueous control recorded the lowest pigment content. In Fe-deficient soil, 60% MLB resulted in maximum chlorophyll (5 mg/g) and carotenoids (6.8 mg/g), outperforming the aqueous control. Under salinity, 60% MLB again recorded the highest pigment levels (4.8 and 6.3 mg/g), whereas 100% MLB and the aqueous control showed the lowest chlorophyll values. These results confirm that moderate MLB concentrations, particularly 60%, significantly enhance pigment production under stress. Figure 14B shows that MLB notably improved carbohydrate, protein, and amino acid content. Under normal conditions, 20% MLB increased carbohydrates (31.1 mg/g), proteins (23.1 mg/g), and amino acids (16.88 mg/g) by 193, 121, and 116% over the aqueous control. *Azospirillum* also performed strongly in these parameters. Under Fe deficiency, 60% MLB boosted carbohydrates (27.1 mg/g), proteins (15.42 mg/g), and amino acids (20.98 mg/g), while *P. fluorescens* recorded similarly elevated biochemical levels. Under salinity, 60% MLB produced the largest increases across all parameters, with carbohydrates, proteins, and amino acids rising by 74, 94, and 61% over the aqueous control. Both bioinoculants significantly enhanced metabolic content under stress, making them strong alternatives or supplements to chemical inputs. Figure 15 indicates distinct phenolic responses across treatments. Under normal conditions, phenols declined from 10.6 mg/g at 20% MLB to 6.3 mg/g at 100% MLB and 5.3 mg/g in the aqueous control. Under Fe deficiency, phenols increased by 64% at 60% MLB compared to the aqueous control, and by 67% under salinity (18.56 mg/g vs. 11.11 mg/g). Chemical controls produced higher phenols than the aqueous treatment, while *Azospirillum* and *P. fluorescens* matched 60% MLB in stress-induced phenol accumulation. Supplementary Figure S6 highlights SOD and catalase activity patterns. Under normal conditions, 20% MLB generated the highest SOD (49.63 units/mg), a 60% improvement over the aqueous control, and the greatest catalase activity (9.97 units/mg). In Fe-deficient and saline conditions, 60% MLB showed maximum SOD activity (53 and 46% increases) and the highest catalase levels (18.77 and 19.83 units/mg), closely followed by *P. fluorescens*. Across all environments, the aqueous control consistently showed the lowest antioxidant activity. These findings underscore the role of MLB particularly 20% under normal and 60% under stress in strengthening enzymatic defenses against oxidative stress.

### 3.6 Elemental composition analysis of plants

Elemental composition analysis of tomato, and paddy plants revealed distinct nutrient profiles under normal, iron-deficient, and saline growth conditions (Table 3). Under normal conditions, in untreated controls, nitrogen (N) concentrations ranged from 2.85% in tomato to 3.62% in paddy, while leaf iron (Fe) content varied from 155 mg/kg in tomato to 150.4 mg/kg in paddy treated with 20% MLB, with sodium (Na) remaining low across crops. Application of 20% MLB and other amendments (Urea, *Azospirillum*) resulted in marked increases in N and Fe concentrations across all crops, with 20% MLB treated plants registering the highest Fe accumulation. These findings indicate that MLB treatments enhance the uptake and assimilation of essential nutrients compared to untreated controls, with Na levels consistently low, indicating minimal salinity stress under normal conditions. Under iron-deficient conditions, all crops exhibited noticeable reductions in foliar Fe content in control treatments, most prominently in paddy (46.33 mg/kg Fe) alongside modest decreases in N concentration. Biofertilizer application (60% MLB) significantly ameliorated Fe deficiency, elevating Fe concentrations to 132.21 mg/kg in tomato, and 146.26 mg/kg in paddy, maintaining the levels considerably higher than untreated controls. These results highlight the potential of MLB in restoring Fe nutrition and supporting N uptake under conditions of iron scarcity. Moreover, the increase in leaf N under MLB treatment reflect active biological N₂ fixation by the MSR-1 nif gene cluster. The nif gene cluster in MSR-1 encodes nitrogenase, which catalyzes ATP-dependent N_2_ reduction to NH_3_ under microaerophilic conditions typical of the rhizosphere oxic–anoxic transition zone where MSR-1 preferentially colonizes. The observed increase in plant nitrogen content provides phenotypic evidence of active biological nitrogen fixation by MSR-1. Additionally, the restoration of foliar Fe under iron-deficient conditions reflects fur/feoB-regulated iron mobilization and magnetosome-derived Fe_3_O_4_ bioavailability. Furthermore, elemental analysis under saline conditions showed increased sodium accumulation in control plants, most notably in tomato (0.76%) and paddy (0.58%), indicating salinity-induced stress. Nitrogen content declined in all control samples, with paddy exhibiting the lowest value (1.14%). Lower N levels under salinity likely result from impaired plant N assimilation and partial inhibition of nitrogenase activity under high ionic stress. In contrast, biofertilizer treatments (60% MLB, *Azospirillum*, and *P. fluorescens*) mitigated the negative impacts of salinity. MLB applications resulted in improved leaf N concentrations and moderate control of Na accumulation, with tomato and paddy exhibiting substantial recovery in N levels (up to 3.09 and 2.82%, respectively). Collectively, these findings highlight the effectiveness of MLB in improving nutrient acquisition and mitigating salt stress in crops underscoring its value for sustainable crop management in challenging agroecological contexts.

**Table 3:** Effect of biofertilizer and conventional treatments on leaf nitrogen, iron, and sodium content of tomato, and paddy under normal, iron-deficient, and saline conditions.

Condition	Normal condition					
Crop	Tomato			Paddy		
Parameters	*N* (%)	Fe (mg/kg)	Na (%)	*N* (%)	Fe (mg/kg)	Na (%)
Control	2.85	60	0.17	3.62	69.27	0.17
20% MLB	4.35	155	0.1	4.81	150.4	0.12
Urea	4	112.15	0.18	4.63	110.33	0.19
*Azospirillum*	4.2	140.89	0.13	4.79	145.89	0.13

Condition	Iron-deficient condition			
Crop	Tomato		Paddy	
Parameters	*N* (%)	Fe (mg/kg)	*N* (%)	Fe (mg/kg)
Control	2.8	48.83	2.05	46.33
60% MLB	3.61	132.21	3.97	146.26
Fe-EDTA	3.05	121.43	3.04	124.46
*P. fluorescens*	3.2	130.98	3.26	140.09

Condition	Saline condition			
Crop	Tomato		Paddy	
Parameters	*N* (%)	Na (%)	*N* (%)	Na (%)
Control	1.95	0.76	1.14	0.58
60% MLB	3.09	0.5	2.82	0.4
Gypsum	2.76	0.59	1.19	0.44
*Azospirillum*	3	0.51	2.71	0.41
*P. fluorescens*	2.88	0.55	2.05	0.42

### 3.7 Soil physiochemical properties analysis

The physicochemical properties of the experimental soils were analysed before and after treatment under both iron-deficient and saline conditions, and the results are summarized in Table 4. The soils used were garden soil mixed with red soil for tomato, and clay soil for paddy.

**Table 4:** Physicochemical properties of soils (iron-deficient and saline) before and after treatment under tomato, and paddy cultivation compared with basic desired levels.

Parameters	Iron-deficient soil				Saline soil				Basic desired level
	Before treatment		After treatment		Before treatment		After treatment		
Crop	Tomato	Paddy	Tomato	Paddy	Tomato	Paddy	Tomato	Paddy	
Soil type	Garden soil + Red soil	Clay soil	Garden soil + Red soil	Clay soil	Garden soil + Red soil	Clay soil	Garden soil + Red soil	Clay soil	
pH	7.5	7.7	7.1	7.3	7.9	7.9	7.5	7.5	6.5–7.5
EC (dS/m)	0.5	0.4	0.7	0.54	2.45	6.1	1.87	5.06	0.5–2
OM (%)	2.9	2.7	3.9	3.5	1.8	1.51	3.55	3.73	1.5–5
NO₃^−^N (ppm)	38.63	36.95	54	57.5	29.75	26.1	44.14	57.64	20–100
Fe (ppm)	3.11	3.68	7.5	8.01	6.8	7.04	7.2	7.9	05–20
P (ppm)	20.21	21.35	19.72	22.53	15	12.54	12.3	12	15–22 for alkali soil
K (ppm)	199.1	193.22	186.41	175.41	175	170.5	178.16	177.72	100–300
Ca (ppm)	2,050	2,022	2,065	2,039	2,070.16	2,088.24	2,070	2,083	2,000–3,000
Mg (ppm)	144.3	130	145.98	135	156	151	160.05	160.4	120–1,500
S (ppm)	13	12.8	13.9	13.7	27.6	26.5	27.8	26.7	10–40
Zn (ppm)	1.2	1.4	1	1.3	1.8	1.62	1.8	1.6	1–5
Mn (ppm)	13.75	15	13	12.99	12.5	10.43	11	17	5–50
Cu (ppm)	0.5	0.6	0.5	0.2	1.2	1.5	1.3	1.5	0.2–5
B (ppm)	0.33	0.4	0.3	0.5	0.31	0.34	0.35	0.38	0.2–2
Cl (ppm)	70	50.38	55	48.9	980	927.38	564	542.61	10–1,000

Before treatment with MLB, soils supporting tomato, and paddy under iron-deficient conditions exhibited pH values within the neutral range (7.2–7.7), with organic matter content ranging from 1.7 to 2.9%. Electrical conductivity (EC) was generally low (0.4–1.6 dS/m), consistent with non-saline environments. Available iron levels were notably limited (3.11–3.68 mg/kg), with nitrate nitrogen and available phosphorus levels relatively moderate (34.5–38.63 mg/kg and 20.21–45.3 mg/kg, respectively). In contrast, exchangeable potassium, calcium, and magnesium levels were within agronomically desirable ranges. Post-treatment analysis in iron-deficient soils revealed significant improvements in several physicochemical metrics. Notably, available iron content increased significantly, reaching up to 7.5 mg/kg in tomato soil, and 8.01 mg/kg in paddy-grown clay soil. There was also an appreciable boost in organic matter (up to 3.9% in tomato-amended soils) and nitrate nitrogen content, particularly in garden soil treatments up to 54 mg/kg and clay soil 57.5 mg/kg. EC values remained within acceptable limits, and essential macronutrients (P, K, Ca, Mg) and micronutrients (Zn, Mn, Cu, B, S) were largely maintained or improved, while chloride levels remained below toxicity thresholds, ensuring overall soil health and fertility.

In saline soils, initial EC values were considerably elevated, especially in paddy-supporting clay soils (up to 6.1 dS/m), indicative of substantial salinity stress. Organic matter was marginal (1.51–1.8%), and available iron (6.1–7.04 ppm) was modest. However, phosphorus (10–15 mg/kg) were near the lower acceptable range, while potassium (120–175 mg/kg), calcium (2070.16–2088.24 mg/kg), magnesium (151–720 mg/kg), sulfur (25.97–27.6 mg/kg), and micronutrients such as Zn, Mn, Cu, and B were within desirable limits. However, chlorine was very high (927.38–987 mg/kg), reflecting strong saline conditions. Following the amendment, EC generally decreased (to as low as 1.87–1.94 dS/m in garden soils) and organic matter increased (up to 3.73%). Nitrate nitrogen levels (44.14–57.64 mg/kg) were elevated post-treatment across crops and available Fe (7.2–7.9 mg/kg) and phosphorus (10.2–12.3 mg/kg) improved slightly, while potassium (129.88–178.16 mg/kg), calcium (2070–2083 mg/kg), and magnesium (160.05–728.2 mg/kg) remained stable within optimum ranges. Chloride accumulation (542.61–774.62 mg/kg), though high in saline environments, remained within permissible bounds after intervention. These results demonstrate the efficacy of the MLB treatment regimens in restoring nutrient balance, enhancing organic matter content, and mitigating the effects of iron deficiency and salinity, thereby supporting optimal plant growth and soil sustainability.

## 4 Discussion

The results of the present study demonstrate that MSR-1, applied as a liquid biofertilizer (MLB), exhibits robust colonization and pronounced positive effects on growth, yield, biochemical parameters, nutrient uptake, and soil health across diverse crops (tomato, paddy), under both optimal and stress conditions. The morphological and physiological characterization of MSR-1 in this study corroborates and extends prior findings in the literature, confirming the bacterium’s distinctive spiral shape, gram-negative nature, and microaerophilic growth preference. The light microscopy and SEM images displaying small spiral cells with smooth surfaces align with earlier detailed morphological descriptions (Lefèvre and Bazylinski, 2013; Leão et al., 2020). Furthermore, the observed microaerobic growth rings near the oxic-anoxic interface are characteristic of MSR-1’s niche, consistent with its adaptation for magnetosome synthesis under low oxygen conditions (Le Nagard et al., 2018). Moreover, the documented growth curve, with cell counts peaking on day 10 before decline, are comparable to growth profiles reported under optimized microaerobic conditions, underscoring the importance of controlled oxygen and iron availability for stable culture (Mira et al., 2022). This lends weight to the recognized sensitivity of MSR-1 to cultivation parameters affecting magnetosome biomineralization and cellular viability (Yan and Xing, 2018).

In addition to this, the SEM-based evidence of MSR-1’s successful colonization of root surfaces in tomato, and paddy confirms its rhizosphere competence and broad host compatibility, consistent with previous studies emphasizing bacteria associative interactions with crop roots (Polanská Nebeská et al., 2026). The bacterial clusters along epidermal surfaces and adherence to leaf trichomes without structural damage indicate not only colonization but potentially beneficial biofilm formation, supporting nutrient exchange and stress mitigation (Kumar et al., 2025). Despite its microaerophilic nature, MSR-1 can transiently survive on tomato and paddy leaves by utilizing protective micro-niches and biofilm-like clustering to buffer against oxygen and desiccation. Short-term oxidative stress tolerance is likely supported by cellular antioxidant systems and magnetosome-associated iron. Rather than actively replicating, the bacteria likely enter a stress-tolerant, metabolically reduced state that maintains plant-growth-promoting potential. This transient persistence in the phyllosphere mirrors documented behavior in other non-native beneficial microbes applied under field conditions. For example, Ajijah et al. confirmed that biofilm formation protects bacteria against desiccation and oxidative stress on plant surfaces (Ajijah et al., 2023). Gayathry et al. documented that extracellular polysaccharides provide desiccation protection in phyllosphere microbes (Gayathry et al., 2024). Moreover, the colonization patterns align with the binding and aggregation dynamics commonly described for plant growth-promoting rhizobacteria (PGPR), reinforced by metabolic compatibility and signaling molecules detected in root exudates. The HR-LCMS profiles of tomato and paddy root exudates revealing chemoattractants such as quinic acid, tryptophan, quercetin, strigolactones, and glucosinolates strongly suggest a chemical basis for selective microbial recruitment (Zhang et al., 2023). These metabolites are recognized for shaping the rhizosphere microbiome, promoting symbiotic and associative interactions essential for biofertilizer efficacy.

Additionally, significant increases were observed in shoot and root lengths, biomass, fruit number, and fruit weight in tomato and paddy treated with MLB under normal, iron-deficient, and saline conditions. Under saline conditions, iron bioavailability is further reduced due to competition between Na^+^/Cl^−^ ions and Fe^2+^ at root uptake sites, creating a co-occurrence of salt stress and iron deficiency. In the present study, MLB application significantly improved growth parameters under saline conditions across all three crops, suggesting a specific interaction between MSR-1 and the saline stress environment. The enhanced performance of MLB under salinity may be attributed to the unique iron biomineralization capacity of MSR-1, wherein magnetosome-bound iron provides a direct and readily available iron source to roots, bypassing the ionic competition characteristic of saline soils. Additionally, MSR-1 inoculation appears to prime the plant’s antioxidant defense system, as evidenced by the elevated phenolic content and antioxidant enzyme activity observed in MLB-treated plants under saline conditions. The observed dose–response shift, wherein 60% MLB outperformed 20% MLB specifically under saline conditions, further suggests that a higher bacterial load is necessary to sustain effective rhizospheric colonization and plant growth promotion under ionic stress. These findings collectively indicate that MSR-1 mitigates salinity stress through complementary mechanisms including direct iron supplementation, antioxidant induction, and osmotic adjustment, rather than functioning as a generic growth promoter. Such parallel findings from recent biofertilizer research demonstrate microbial inoculants’ capacity to enhance plant growth under multiple abiotic stresses (Bhavya et al., 2017). The comparative advantage of 20% MLB under normal conditions and 60% MLB under stress aligns with dose-dependent biofertilizer activity. Under normal conditions, 20% MLB was sufficient for effective rhizosphere establishment whereas, the higher 60% dose was required for iron-deficient and saline conditions. This is probably because osmotic stress and increased ionic strength decrease MSR-1 survival after inoculation, requiring a larger initial cell density to sustain an active rhizosphere population. Furthermore, a larger inoculum level may be favored under iron deficit due to increased competition with native iron-chelating microbes. Increased phenolics and antioxidant enzyme activity at 60% MLB under stress circumstances further point to potential (Induced Systemic Resistance) ISR-like priming, which could need a threshold microbial density to produce enough elicitor signals. These mechanisms are still theoretical and need more research. For example, Anli et al. demonstrated that dual-inoculation of mycorrhizal fungi and plant growth-promoting rhizobacteria significantly enhanced biomass under water deficit conditions (Anli et al., 2020). Furthermore, Alotaibi et al. found that AMF biofertilizer increased grain yield by 17.27–17.33% under drought stress (Alotaibi et al., 2024). Further, enhanced chlorophyll and carotenoid contents closely correlate with improved photosynthetic capacity and stress tolerance, consistent with prior observations in PGPR-treated crops (Shabaan et al., 2022; Chang et al., 2024). Moreover, the elevated carbohydrate, protein, and amino acid levels under MLB treatments substantiate improved metabolic status, corroborated by increased phenolic content and antioxidant enzyme activities (superoxide dismutase and catalase), which jointly mediate oxidative stress alleviation in plants facing iron deficiency and salinity. Multiple studies demonstrate these relationships under salt stress such as Xu et al. reported PGPR increased chlorophyll content by 27.17% and enhanced antioxidant defense under salinity in maize (Xu et al., 2025). This dual enzymatic and non-enzymatic antioxidant enhancement reflects a synergistic plant-microbe interaction promoting abiotic stress resilience.

Data on paddy reveal similar trends of growth promotion and biochemical enhancement with MLB treatment, particularly at 20% for normal and 60% for stressed soils, echoing observations in tomato and providing evidence for broad-spectrum applicability across crop species. Furthermore, higher tiller and panicle numbers reflect improved vigor and yield stability under stress. The consistent dose–response patterns across parameters are mechanistically expected, as microbial inoculants act systemically through shared physiological pathways, with the optimal dose shift from 20 to 60% under stress reflecting increased colonization requirements under adverse conditions. Directional uniformity across parameters thus reflects coherent biological action, while variation in absolute response magnitudes between species and parameters confirms the authenticity of the dataset. MLB application enhanced soil organic matter, nutrient availability, and iron levels while lowering electrical conductivity in saline soils, indicating reduced salinity stress and improved fertility through rhizosphere nutrient cycling and ionic balance. These findings are robust and consistently supported by numerous studies such as Sachin et al. demonstrated that halophilic bio-formulations increased tillers per hill by 12.38% and panicle length by 33.48% in sodic soil (Tomar et al., 2025). Regarding soil improvements, Shan et al. found biofertilizer application notably increased available nitrogen, phosphorus, and potassium while significantly decreasing soil electrical conductivity (*p* < 0.05) (Shan et al., 2023).

Elemental analyses show MLB enhances nitrogen and iron uptake, improving metabolism and stress tolerance, with increased foliar iron and reduced sodium under salinity reflecting MSR-1’s multifunctional genetic capacity. Under Fe-deficient conditions at 60% MLB, foliar iron increased to 132.21 mg/kg (tomato), and 146.26 mg/kg (paddy). The increase in foliar iron concentration indicates that MSR-1 increases iron bioavailability through both active and passive processes. Active process is mediated by iron-regulatory systems such as fur and feoB, which promote iron acquisition in low-iron environments. Moreover, passive process is mediated by releasing the iron stored in magnetosomes (Fe_3_O_4_) during bacterial cell death and made available to plants. The significant increase in foliar iron suggests that active Fur/FeoB mobilization process played the predominant role. Comparable to strains like *Azospirillum* and *Pseudomonas fluorescens*, MLB performs effectively under both optimal and stress conditions. Overall, MSR-1 is a promising, stress-resilient biofertilizer that improves plant growth and soil health, though large-scale production and field validation require further study.

## 5 Conclusion

This study evaluated the multifunctional potential of MSR-1 as a biofertilizer (MLB) for tomato and paddy under normal, iron-deficient, and saline conditions. Morphological and ultrastructural analyses confirmed MSR-1’s spiral cell structure, while soil survival studies showed peak metabolic activity on day 10. SEM assays demonstrated successful, non-damaging colonization of MSR-1 on roots and leaves. Further, HR-LCMS of root exudates revealed chemoattractants supporting plant–microbe compatibility. Moreover, pot trials indicated concentration-dependent effects demonstrating 20% MLB was optimal under normal conditions, while 60% MLB excelled under Fe deficiency and salinity conditions. Foliar iron increased to 132.21 mg/kg (tomato) and 146.26 mg/kg (paddy), while salinity stress mitigation included reduced sodium (0.4–0.50%) and improved nitrogen uptake (3.09% tomato, 2.82% paddy). Soil analyses confirmed enhanced organic matter, nitrate nitrogen, and available Fe, with reduced electrical conductivity in saline soils. Overall, MSR-1–based biofertilizers may represent a transformative tool for sustainable, resource-efficient, and climate-resilient agriculture. However, further studies are needed to assess long-term ecological impacts and performance across diverse agroecosystems.

## Data Availability

The original contributions presented in the study are included in the article/Supplementary material, further inquiries can be directed to the corresponding author.
