# Supplementary material for: Magnetotactic bacteria as biostimulants for enhancing the growth and yield of tomato and paddy under abiotic stress
Source: Front Microbiol. 2026 Jun 26;17:1848546. doi: 10.3389/fmicb.2026.1848546 (PMC13350338; doi:10.3389/fmicb.2026.1848546)

Table S1 Treatment groups used to study the effect of MLB under normal, iron deficiency, and saline stress conditions

MLB under normal conditions	MLB under iron deficiency	MLB for salinity stress
Group 1: 20% MLB	Group 1: 20% MLB	Group 1: 20% MLB
Group 2: 40% MLB	Group 2: 40% MLB	Group 2: 40% MLB
Group 3: 60% MLB	Group 3: 60% MLB	Group 3: 60% MLB
Group 4: 80% MLB	Group 4: 80% MLB	Group 4: 80% MLB
Group 5: 100% MLB	Group 5: 100% MLB	Group 5: 100% MLB
Group 6: Distilled water	Group 6: Distilled water	Group 6: Distilled water
Group 7: 2 % Urea	Group 7: Fe-EDTA (6%)	Group 7: 0.2 % Gypsum
Group 8: *Azospirillum* (0.5ml)	Group 8:*P. fluorescens* (0.5 ml)	Group 8: *Azospirillum* (0.5ml)
		Group 9:*P. fluorescens* (0.5 ml)

Figure S1. Microaerophilic band of MSR-1 acquired from soil. Sample **(A)** MSGM medium. Sample **(B)** MSGM medium + MSR-1

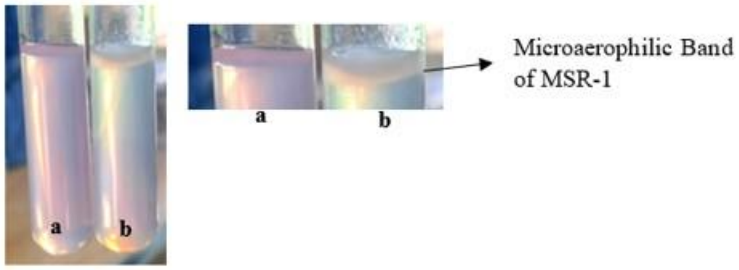

Figure S2. Fragment ion spectra of tomato root exudates showing characteristic peaks at m/z 412.353 and 437.168

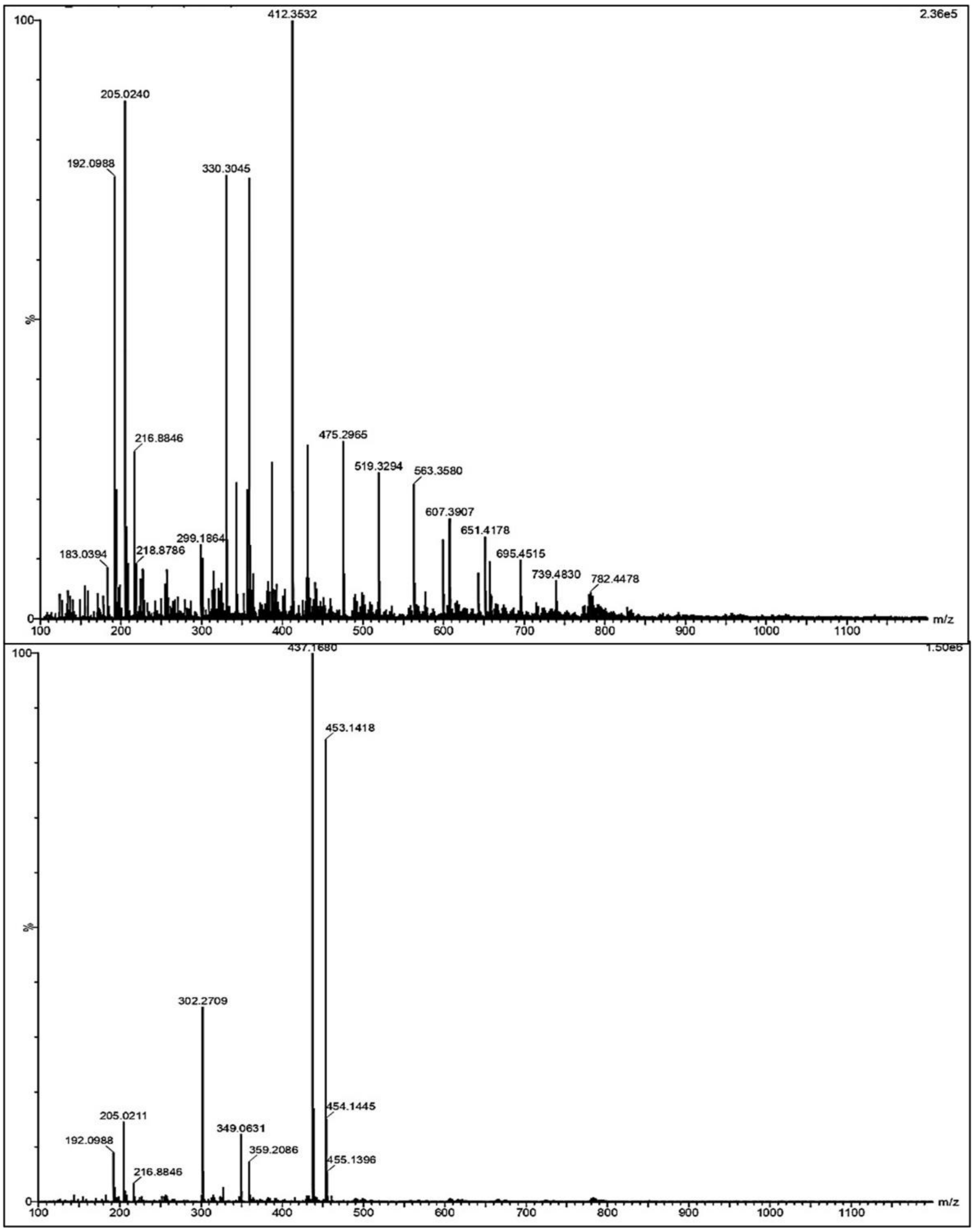

Figure S3. Fragment ion spectra of paddy root exudates with characteristic peaks observed at m/z 389.159 and 437.183

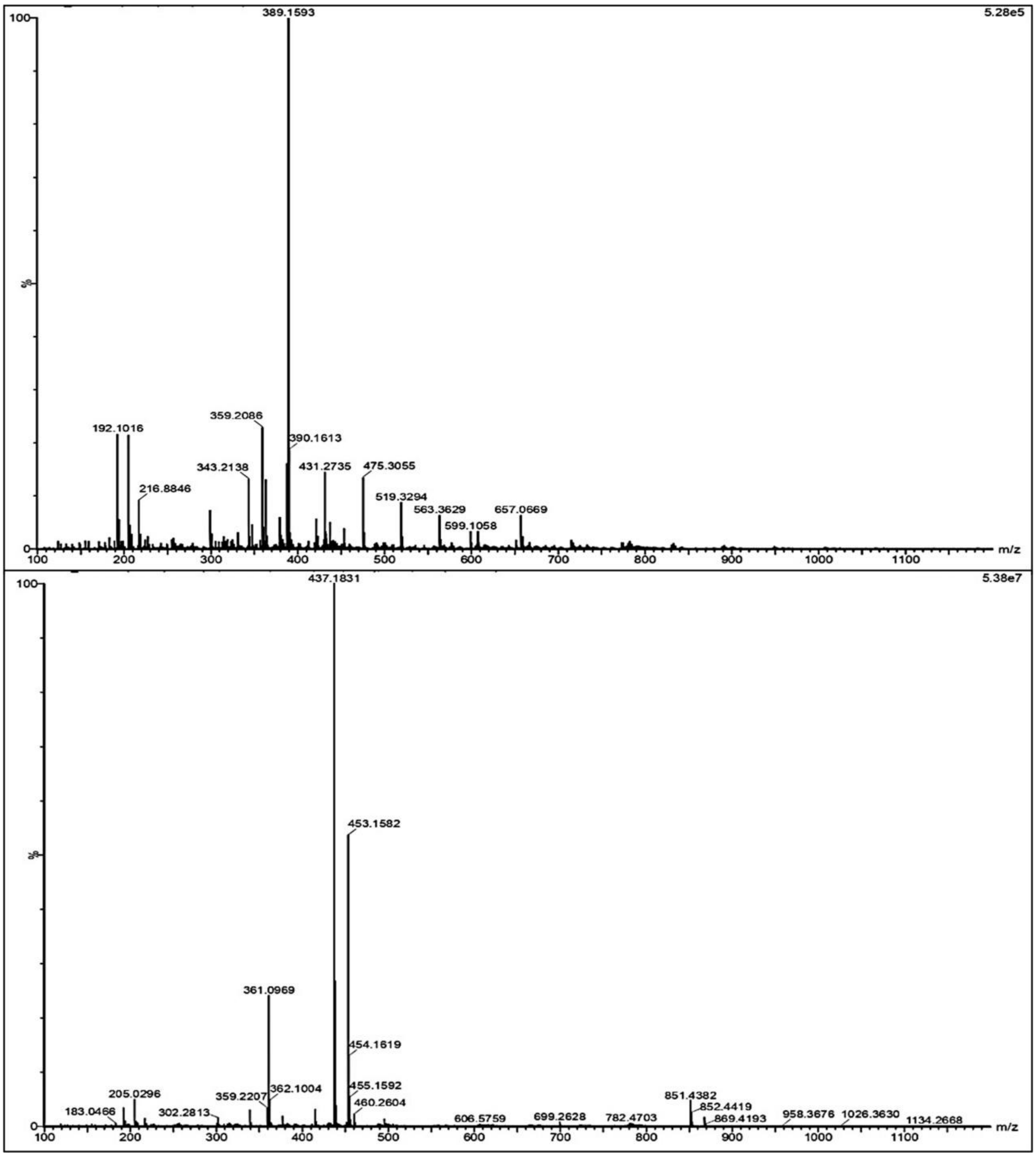

Figure S4. Notable differences in shoot and root length of tomato under normal, iron-deficient, and saline conditions.

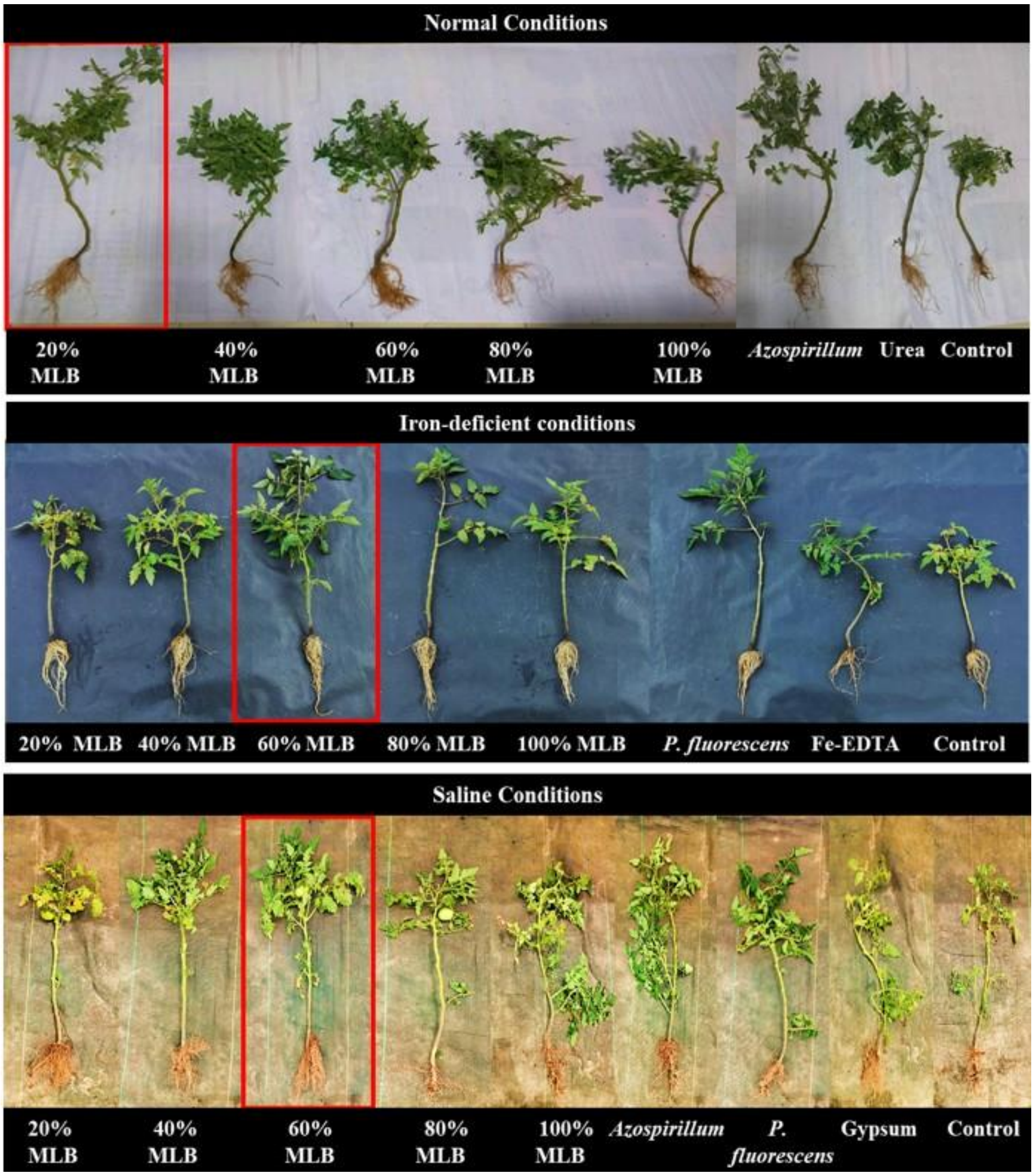

Figure S5. Variation in shoot and root growth of paddy observed across normal, Fe-deficient, and saline conditions

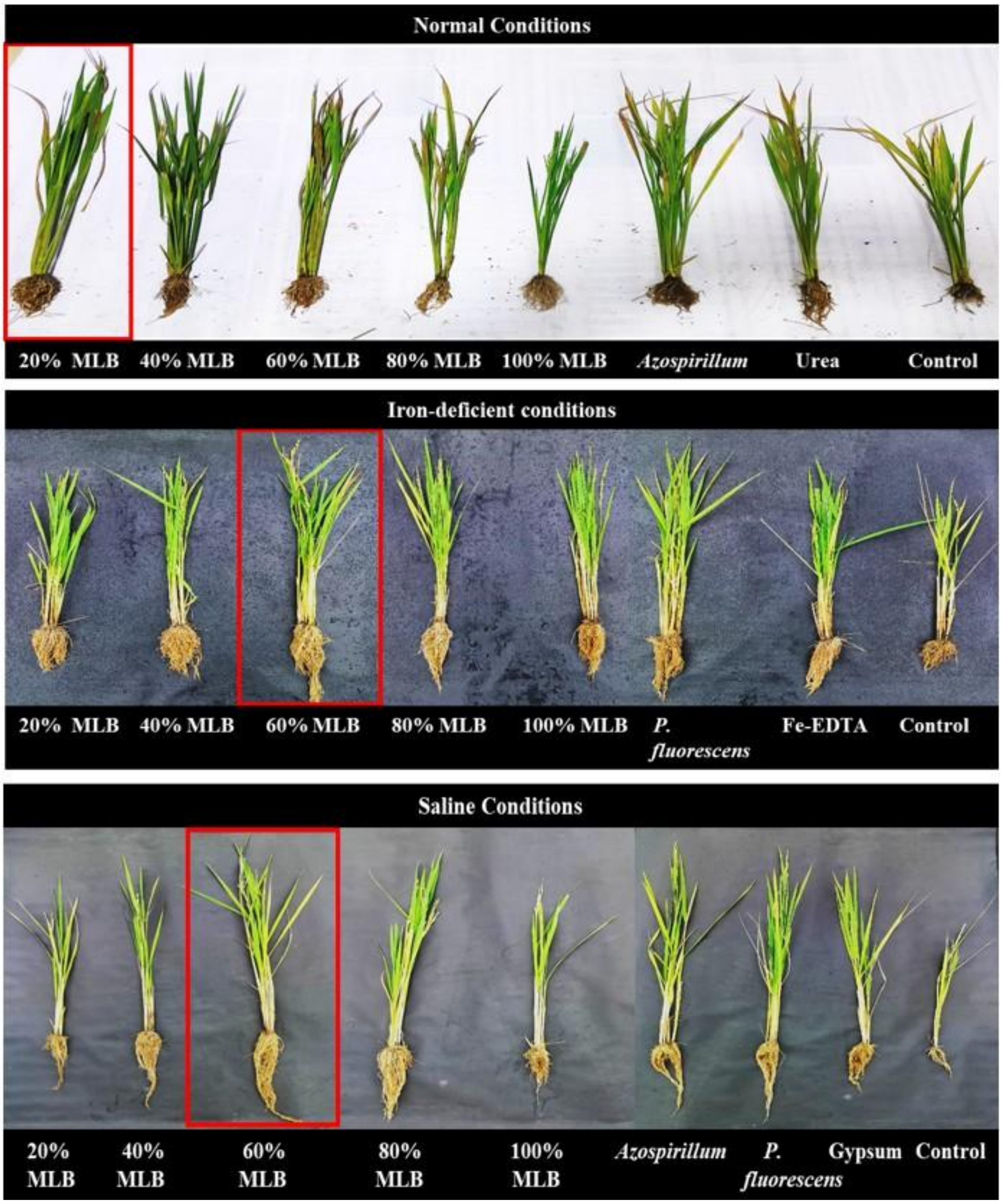

Figure S6. Assessment of the effects of MLB and controls on SOD and catalase of paddy under standard, iron-deficient, and saline growth conditions. Means ± SEM (n = 3); One-way ANOVA ($p < 0.05$).

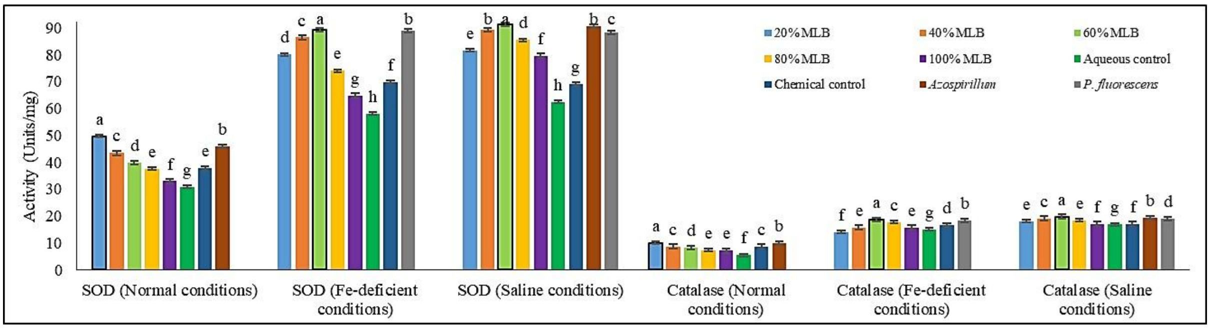

Supplement: Supplementary file 1 [file Data_Sheet_1.PDF]
